# Partial coherence enhances parallelized photonic computing

**DOI:** 10.1038/s41586-024-07590-y

**Published:** 2024-07-31

**Authors:** Bowei Dong, Frank Brückerhoff-Plückelmann, Lennart Meyer, Jelle Dijkstra, Ivonne Bente, Daniel Wendland, Akhil Varri, Samarth Aggarwal, Nikolaos Farmakidis, Mengyun Wang, Guoce Yang, June Sang Lee, Yuhan He, Emmanuel Gooskens, Dim-Lee Kwong, Peter Bienstman, Wolfram H. P. Pernice, Harish Bhaskaran

**Affiliations:** 1 Department of Materials, University of Oxford, Oxford, UK; 2 Institute of Microelectronics, Agency for Science, Technology and Research (A*STAR), Singapore, Singapore; 3 Kirchhoff-Institute for Physics, Heidelberg University, Heidelberg, Germany; 4 Center for NanoTechnology, University of Münster, Münster, Germany; 5 Photonics Research Group, Ghent University – imec, Ghent, Belgium

**Keywords:** Applied optics, Nanophotonics and plasmonics

## Abstract

Advancements in optical coherence control^1–5^ have unlocked many cutting-edge applications, including long-haul communication, light detection and ranging (LiDAR) and optical coherence tomography^6–8^. Prevailing wisdom suggests that using more coherent light sources leads to enhanced system performance and device functionalities^9–11^. Our study introduces a photonic convolutional processing system that takes advantage of partially coherent light to boost computing parallelism without substantially sacrificing accuracy, potentially enabling larger-size photonic tensor cores. The reduction of the degree of coherence optimizes bandwidth use in the photonic convolutional processing system. This breakthrough challenges the traditional belief that coherence is essential or even advantageous in integrated photonic accelerators, thereby enabling the use of light sources with less rigorous feedback control and thermal-management requirements for high-throughput photonic computing. Here we demonstrate such a system in two photonic platforms for computing applications: a photonic tensor core using phase-change-material photonic memories that delivers parallel convolution operations to classify the gaits of ten patients with Parkinson’s disease with 92.2% accuracy (92.7% theoretically) and a silicon photonic tensor core with embedded electro-absorption modulators (EAMs) to facilitate 0.108 tera operations per second (TOPS) convolutional processing for classifying the Modified National Institute of Standards and Technology (MNIST) handwritten digits dataset with 92.4% accuracy (95.0% theoretically).

## Abstract (web summary)

Two photonic platforms using a convolutional processing system with partially coherent light sources is shown to boost computing parallelism, demonstrated using the classification of gaits of patients with Parkinson’s disease and the MNIST handwritten digits dataset.

## Main

Over the past century, notable progress in optical coherence control has enabled the generation of light with linewidth ranging from tens of terahertz (THz) to less than 1 kilohertz (kHz). This enhanced control has revolutionized light sources, from fluorescence^1^, light-emitting diodes (LEDs)^2^ and lasers^3,12^ to distributed-feedback lasers^4^ and stabilized continuous-wave lasers^5,13^, laying the foundation for numerous transformative applications, such as long-haul optical-fibre communications^6^, LiDAR^7^, optical coherence tomography^8,14^ and so on. Despite the challenges in stabilizing and maintaining high optical coherence, researchers have sought to make use of the superior properties of coherent light by using partially coherent light in combination with post-processing reconstruction methods as a compromised solution^9,15,16^. Taking a more direct approach, many studies have aimed to generate more coherent light from incoherent light sources^17,18^, with a recent achievement in obtaining spatio-temporal coherence with an incoherent white-light source for coloured vortex-beam generation using miniaturized spiral phase plates integrated with structural colour filters^10^. As a result, increasing optical coherence has become a guiding principle for improving the performance and functionalities of cutting-edge optical devices and systems.

Deep learning has made a great impact on various fields^19–22^, with two recent highlights being GPT-4 and Midjourney. The success of deep learning relies on training huge artificial neural networks with billions of trainable parameters, necessitating the doubling of hardware-data processing capability every 3.5 months^23^. To keep up with this exponentially growing need for processing capability, photonic convolutional processing is believed to be a key to hardware-based artificial intelligence (AI) accelerators^24–26^. Photonic processors can access a wide bandwidth of tens of THz by exploiting wavelength-division multiplexing and eliminate capacitive delay and charge/discharge energy dissipation, as photons require no potential difference to transit^27^. Various system architectures for photonic convolutional processing have been proposed, all using coherent light sources in accordance with the guiding principle. Coherent nanophotonic circuits distribute light from a single coherent light source to the inputs of a Mach–Zehnder interferometer (MZI) array^11,28–30^. Operating these circuits requires the precise control of numerous phase shifters to ensure the desired coherent interference in the circuit. A broadcast-and-weight protocol based on cascaded microring resonator (MRR) arrays has been demonstrated^31–34^. The optical input is created by multiplexing coherent light across several wavelengths, with each wavelength being weighted by a corresponding tunable MRR of varying radii and combined in a common bus waveguide. Convolutional processing based on the broadcast-and-weight protocol requires precise control over a substantial number of MRRs, and one convolution operation on an *N*-dimensional vector requires *N* distinct coherent lights at different wavelengths. On-chip diffractive optical neural networks have been showcased, performing matrix-vector multiplication (MVM) operations within an ultra-compact footprint through coherent interference of accurately controlled diffractive light^35,36^. To achieve in-memory photonic convolutional processing, which eliminates the need for data movement between the memory and photonic processors, a photonic tensor core incorporating phase-change-material photonic memories was proposed and demonstrated^37,38^. A silicon nitride MRR was pumped by a coherent laser to generate a frequency comb. In a photonic tensor core consisting of *N* inputs and *M* outputs, *N* different wavelength components must enter their corresponding inputs to prevent measurable interference effects, which could result in unwanted intensity fluctuations. Data carried by each input coherent light at different wavelengths are weighted by the phase-change-material photonic memories and combined in a common bus.

Here we demonstrate that decreasing optical coherence can enhance photonic convolutional processing. We present a photonic convolutional processing system that takes advantage of decreased temporal coherence, hereafter referred to as a partially coherent system, to boost processing parallelism without substantially sacrificing accuracy and potentially enable large-scale photonic tensor cores. This approach eliminates the need for precise control of numerous phase shifters or MRRs and eases the requirements for stringent feedback control and thermal management by using partially coherent light sources. We showcase the broad applicability of partial coherence processing in two photonic platforms for computing applications: first, we conduct parallel convolutional processing with a 3 × 3 photonic tensor core using phase-change-material photonic memories for classifying the gaits of ten patients with Parkinson’s disease and achieve an accuracy of 92.2%; and second, we implement a high-speed 0.108 TOPS convolution processor using a 9 × 3 silicon photonic tensor core with embedded EAMs for vector encoding and weight setting, combined with on-chip photodetectors to classify the MNIST handwritten digits dataset with an accuracy of 92.4%.

## Partial coherence as key to enhanced parallelism

**Fig. 1:**
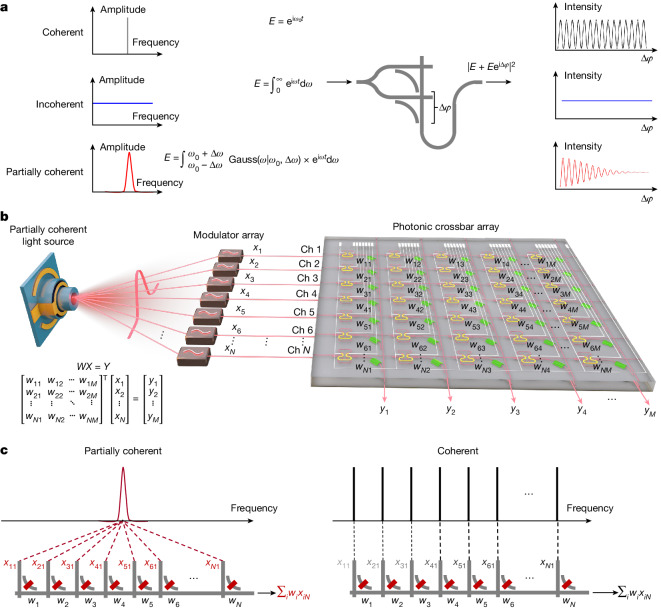
Concept of partial-coherence-enhanced parallelized photonic computing. **a**, Intensity fluctuation with respect to an increasing phase difference Δ*φ* in a single computation unit cell for multiply-and-accumulate operations when a coherent, incoherent or partially coherent light source is used. Gauss(*ω*|*ω*_0_, Δ*ω*) represents a Gaussian distribution with a mean value of *ω*_0_ and standard deviation Δ*ω*. **b**, Working principle of parallelized photonic computing using partially coherent light. **c**, *N*-fold enhancement in parallelism. *N* is the dimension of input vectors.

State-of-the-art photonic tensor cores use coherent light sources, such as distributed-feedback lasers and frequency combs, for computation. A generalized unit cell to perform multiply-and-accumulate operations is shown in Fig. 1a. Light is equally split into two arms, with multiplication performed in each arm and the multiplication results summed in a common bus waveguide. The fluctuation in transmission intensity resulting from fluctuation of phase difference (Δ*φ*) is determined by the coherence property of input light. Figure 1a illustrates the dependence of intensity fluctuation on phase difference. For a coherent light source at a fixed single frequency ${E={\rm{e}}}^{{\rm{i}}{\omega }_{0}t}$, the output intensity |*E* + *E*e^iΔ*φ*^|^2^ changes sinusoidally with phase difference. For an idealized incoherent light source that spans the entire frequency range, the output is unaffected by phase fluctuation. A partially coherent light source provides immunity to phase fluctuations but only makes use of a limited optical bandwidth, which makes it compatible with wavelength-division multiplexing. Partially coherent light progressively loses dependency on phase fluctuation as the phase difference increases. For small phase differences, the intensity fluctuates, resembling coherent light; at larger phase differences, the intensity remains stable, as in the case of incoherent light.

A system that makes use of partially coherent light for parallelized photonic computing is proposed in Fig. 1b. The light source does not need to be a coherent light source that demands precise feedback control and thermal management. Instead, we can use a superluminescent diode (SLED)^39,40^ or filtered light from a broadband light source, enabling simpler integration and less stringent circuit management. The partially coherent light is then evenly distributed to *N* input channels, with each channel modulated to generate the input vector (*x*_1_ ⋯ *x*_*N*_)^T^. MVM is performed by the photonic tensor core with weights encoded in the photonic crossbar array. The weighting elements can be any photonic device that enables amplitude modulation, such as the phase-change-material photonic memories or EAMs used here. This partially coherent system offers much higher parallelism when compared to a coherent system. As shown in Fig. 1c, a Gaussian-shaped optical carrier can be sent to all input channels and summed in a bus waveguide, as intensity fluctuation caused by phase fluctuations is eliminated. By contrast, in a coherent system, different input channels should receive optical carriers at distinct wavelengths to avoid intensity fluctuation. Consequently, one MVM operation for input vectors of dimension *N* requires only one optical band when using partially coherent light but consumes *N* optical bands if coherent light is used. The enhancement in parallelism is thus *N*-fold when using partially coherent light as compared to coherent light. This also implies better scalability of the photonic tensor core. The scalability of a partially coherent system will not be limited by the spectral window of photonic components, as the input optical bandwidth does not scale with input vector dimension.

## Coherence properties of light sources

**Fig. 2:**
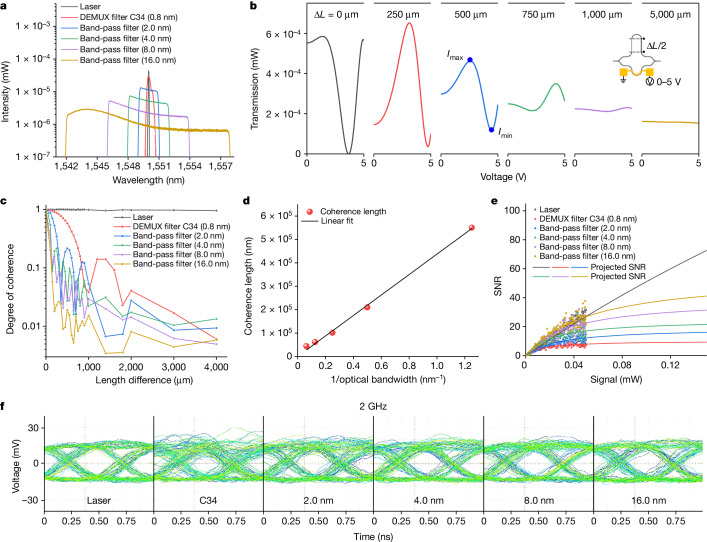
Coherence properties of light sources. **a**, Spectra of investigated light sources with different bandwidths. **b**, Transmission spectra when the relative phase is swept in MZIs with increasing length differences. The input light is 0.8-nm-bandwidth C34 partially coherent light. **c**, Measured degree of coherence. **d**, Coherence lengths extracted from **c** are inversely proportional to optical bandwidth. **e**, Dependence of SNR on signal (intensity received at the photodetector). **f**, Two-GHz eye diagram at 0.05-mW signal. Source Data

Coherent and partially coherent light is generated from a coherent laser and by filtering the amplified spontaneous emission (ASE) of an erbium-doped fibre amplifier (EDFA), respectively. The wavelength spectra of the investigated light sources centred around 1,550 nm are shown in Fig. 2a, including a Gaussian-shaped coherent source with a linewidth narrower than 70 pm, a Gaussian-shaped partially coherent source with 0.8-nm bandwidth filtered by a demultiplexer (DEMUX) on ITU grid channel C34 and four non-Gaussian-shaped partially coherent sources with 2.0, 4.0, 8.0 and 16.0 nm bandwidths filtered by an optical tunable band-pass filter. All light sources are operated in continuous-wave mode. A thermo-optically controlled MZI array with increasing path differences is used to determine the coherence lengths of all lights (Supplementary Fig. 1). The concept proposed in Fig. 1a is verified in Fig. 2b, which illustrates diminishing phase sensitivity with increasing length differences. The degree of coherence, defined by the interference strength $\frac{{I}_{\max }-{I}_{\min }}{{I}_{\max }+{I}_{\min }}$, is extracted from Fig. 2b and presented in Fig. 2c. The degree of coherence of coherent light maintains a level around unity at a large length difference of 4,000 µm. By contrast, that of partially coherent light decreases notably with increasing length differences, with a generally lower degree of coherence accompanied by a wider optical bandwidth. Quantitatively, the coherence length, defined as the length difference for which the degree of coherence decreases to 0.5, is inversely proportional to the optical bandwidth (Fig. 2d), in agreement with theory^41^. A comparison between Gaussian-shaped and non-Gaussian-shaped partially coherent light reveals a negligible difference in the degree of coherence and coherence length (Supplementary Fig. 2).

We investigate the effect of optical bandwidth and noise of filtered ASE on optical modulation. For coherent light, the noise remains at a low level above the system noise floor and exhibits a weak dependence on the intensity received at the photodetector (Supplementary Fig. 3). Conversely, for partially coherent light, the noise increases linearly with the intensity received at the photodetector and is inversely related to optical bandwidth. This observation can be explained by the stochastic properties of an EDFA^42^, which introduce inherently elevated noise levels compared with coherent light. However, this noise can be reduced by increasing the ratio of optical bandwidth to electrical bandwidth. The linearly increasing noise leads to a saturated signal-to-noise ratio (SNR) in partially coherent light (Fig. 2e), implying that coherent light holds an advantage in high-intensity scenarios in which partially coherent light is hampered by a compromised SNR. Nonetheless, in integrated photonic circuits, the signal (intensity received at the photodetector) typically spans from 0.1 µW to 0.1 mW. In this range of interest to many applications, the SNR of partially coherent light from EDFA ASE is not substantially lower compared with that of coherent light. The viable SNR of partially coherent light is verified by measuring 2-GHz eye diagrams at 0.05-mW signal (Fig. 2f). Although the eye diagram of 0.8-nm-bandwidth C34 partially coherent light is ambiguous compared with coherent light, the clarity of the eye diagram markedly improves with an enlarged optical bandwidth, becoming clear at 4.0-nm bandwidth and beyond. At a lower modulation speed of 100 MHz, all eye diagrams are clear (Supplementary Fig. 4).

## Eliminating intensity fluctuation

**Fig. 3:**
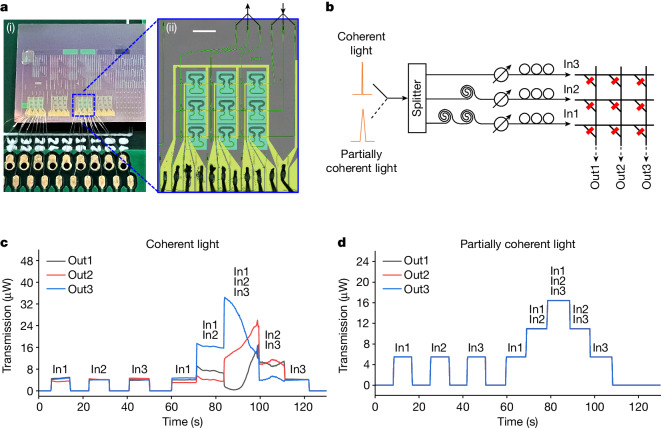
Elimination of intensity fluctuation in a system using partially coherent light. **a**, Optical images of the photonic memory crossbar array. (i) Integrated photonic tensor core. (ii) Zoom-in of the 3 × 3 photonic memory crossbar array. Scale bar, 200 µm. **b**, Schematic of the setup to investigate intensity fluctuation. The partially coherent light is 0.8-nm-bandwidth C34. **c**,**d**, Time trace of transmission with different input channels turned on when using coherent light (**c**) and partially coherent light (**d**). Source Data

The elimination of intensity fluctuation caused by phase fluctuation within a single MZI has been verified in Fig. 2b. When transferring this concept from a single-device level to a system level, we must account for potential complexities and further variables that may influence the stability and reliability of the entire photonic system, requiring further verification at the system level. A photonic tensor core using phase-change photonic memories featuring the architecture proposed in Fig. 1b, hereafter referred to as photonic memory tensor core, is fabricated to perform enhanced parallelized photonic computing. As a proof of concept, the photonic memory crossbar array represents a 3 × 3 weight matrix (Fig. 3a) and the working principle is described in Supplementary Text 1. The weights are encoded in non-volatile phase-change-material photonic memories. Using the pump–probe weight-setting scheme^43^, the non-volatile amplitude modulation enabled by controlling the crystalline state of phase-change-material photonic memories enables 4-bit operations. The maximum transmission change *T*_max_ − *T*_min_ is greater than 20% (Supplementary Fig. 6). The transmission levels *T* are mapped to weights *w* in [−1, 1] by defining $w=\frac{T-\frac{{T}_{\max }+{T}_{\min }}{2}}{\frac{{T}_{\max }-{T}_{\min }}{2}}$. In this work, the mapping is implemented by post-processing on a computer (Methods). This mapping approach can be implemented in hardware using a balanced photodetection scheme (Supplementary Text 2). On the other hand, besides changing the hardware architecture, the neural networks themselves can be modified to adapt to the non-negative nature of photonic computing systems^44^.

Figure 3b presents the schematic of the setup used to investigate the intensity fluctuation when using coherent or partially coherent light (0.8-nm-bandwidth C34). A path difference of 1 m between adjacent inputs is introduced by incorporating 1-m-long fibre delays. This 1-m path difference is substantially longer than the measured coherence length of 550 µm in 0.8-nm-bandwidth C34 partially coherent light, which will effectively eliminate intensity fluctuations. When coherent light is split and directed to three input channels, strong intensity fluctuations are observed (Fig. 3c), resulting from phase fluctuations along the optical paths. On the contrary, when partially coherent light is used, intensity fluctuations are eliminated (Fig. 3d). This immunity of transmission intensity to phase fluctuation is the desired property offered by partially coherent light that will enable higher parallelism. Specifically, using partially coherent light, light in one optical band can be distributed to all input channels to perform MVM operations, allowing for full bandwidth use.

## Parallelized convolution of gait signals from patients with Parkinson’s disease

**Fig. 4:**
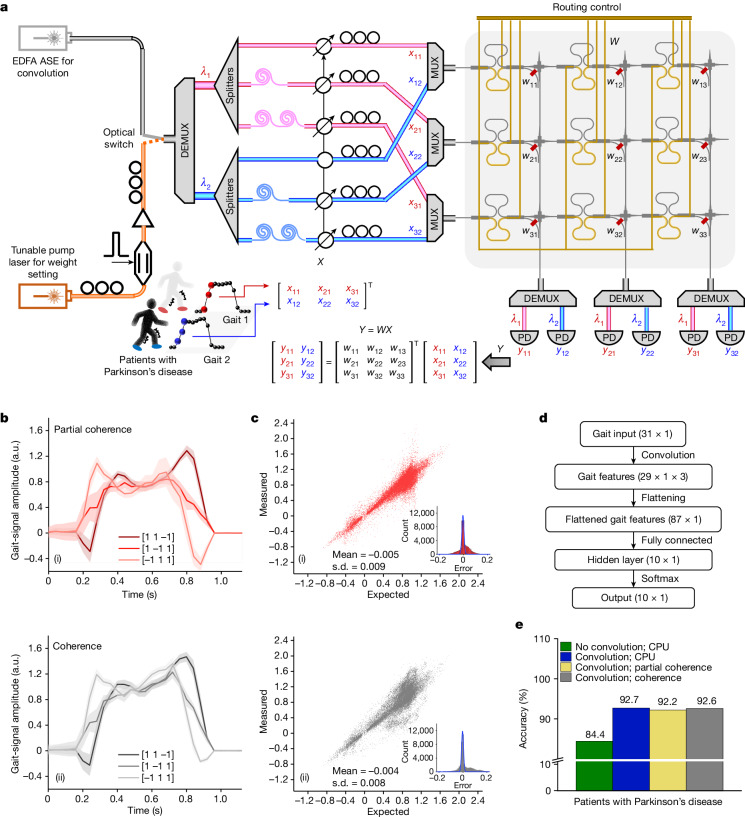
Parallelized convolution of gait signals from patients with Parkinson’s disease. **a**, Schematic of the computing system using the 3 × 3 photonic memory tensor core. The partially coherent lights in use are 0.8-nm-bandwidth C34 and C33. **b**, Convolution results of a typical gait signal from three different kernels when using a partially coherent system in (i) and a coherent system in (ii). All of the convolutions are performed once. The error bands represent the standard deviation of convolution results from 50 gait signals generated by the same patient, showing the variation of gait signals from this patient. **c**, Convolution accuracy of using a partially coherent system in (i) and a coherent system in (ii). A total of 43,500 pairs of expected and measured results are compared in each system. The insets show the Gaussian distribution of normalized errors. **d**, CNN architecture. **e**, Comparison of CNN classification results. a.u., arbitrary units; PD, photodetector. Source Data

As a proof-of-concept example to showcase the capability of partial-coherence-enhanced parallelized photonic computing, we construct a system using the photonic memory tensor core to identify patients with Parkinson’s disease by analysing their gaits. The enhanced parallelism offers a way to simultaneously monitor a large number of patients. The partially coherent light has a bandwidth of 0.8 nm, modulated at 1 kHz. As shown in Fig. 4, gait signals from patients with Parkinson’s disease originally in the form of time series are recorded. The gait signal from patient *j* at time *i* is represented by *x*_*ij*_. *x*_*ij*_ is carried by wavelength *λ*_*j*_ and sent into optical channel *i*. Taking the gait signal from patient 1 for example, the input vector is (*x*_11_, *x*_21_, *x*_31_)^T^ carried by *λ*_1_. The 3 × 3 photonic memory crossbar array defines three kernels of dimension 3 × 1, represented by the weight matrix $$W={\left[\begin{array}{ccc}{w}_{11} & {w}_{12} & {w}_{13}\\ {w}_{21} & {w}_{22} & {w}_{23}\\ {w}_{31} & {w}_{32} & {w}_{33}\end{array}\right]}^{{\rm{T}}}$$. Specifically, the rows of *W* are set to $${\left[\begin{array}{c}1\\ 1\\ -1\end{array}\right]}^{{\rm{T}}}$$, $${\left[\begin{array}{c}1\\ -1\\ 1\end{array}\right]}^{{\rm{T}}}$$ and $${\left[\begin{array}{c}-1\\ 1\\ 1\end{array}\right]}^{{\rm{T}}}$$, performing right-edge extraction, peak suppression and left-edge extraction, respectively. Using an extra wavelength *λ*_2_, the system can perform convolutional processing for patient 2 in parallel. For comparison, the schematic of a coherent system to implement the same convolutional processing is described in Supplementary Text 3. Notably, in the partially coherent system, the same wavelength can enter different input waveguide channels because the intensity fluctuation is eliminated. The ability to enable the same wavelength to enter different input channels provides superior advantages compared with the conventional computing system that uses coherent light. The optical bandwidth is fully used because no further wavelength channels are required to avoid intensity fluctuation. In comparison with our partially coherent system, which uses two wavelengths, a coherent system will require six wavelengths. This advantage scales up with the desired parallelism and the size of the photonic tensor core. For a parallelism of *P* (that is processing gait signals from *P* patients in parallel) and a photonic tensor core with dimension *N* by *M*, the reduction in the required number of wavelengths is (*N* − 1) × *P*.

The convolution results obtained using the partially coherent system are shown in Fig. 4b–e and compared with the results obtained using a coherent system. For a typical gait signal, the desired features are successfully extracted in both computing systems, as shown in Fig. 4b. Theoretical convolution results obtained by a central processing unit (CPU) are presented in Supplementary Fig. 9. The convolution results of all gait signals from all ten patients are presented in Supplementary Figs. 10 and 11. Figure 4c shows the accuracy of the convolution operation. The two computing systems show similar accuracy. The respective errors follow Gaussian distributions and show close mean values and standard deviations. Using these convolution results obtained by the photonic systems, we construct a convolutional neural network (CNN) to identify patients with Parkinson’s disease (Fig. 4d). The CNN is first implemented by a CPU to test the necessity of the convolution layer. Using a convolution layer, the classification accuracy is improved from 84.4% to 92.7% (Fig. 4e). When the convolution layer is implemented by photonic systems, the classification accuracy reaches more than 92.2% in both computing systems, showing a performance close to the CPU implementation. The confusion maps of CNN classification results are presented in Supplementary Fig. 12. The evolution of CNN loss and accuracy with respect to increasing epochs are presented in Supplementary Fig. 13. The partially coherent system achieves similar performance to the coherent system, but with much fewer wavelength channels and less stringent light-source requirements.

## High-speed convolution of MNIST datasets

**Fig. 5:**
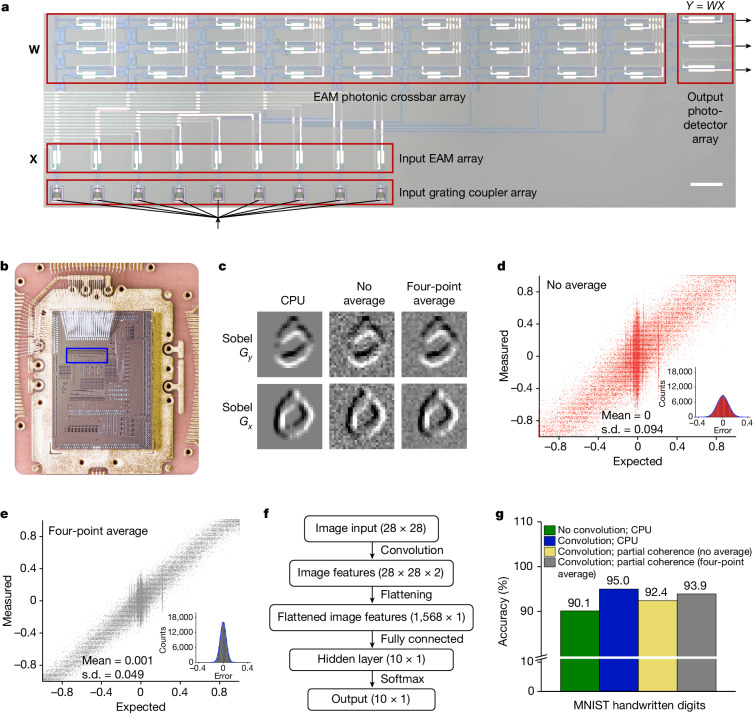
High-speed convolution of the MNIST handwritten digits dataset. **a**, Optical image of the 9 × 3 photonic EAM tensor core. The input light is 8.0-nm-bandwidth partially coherent light. Scale bar, 100 µm. **b**, Optical image of the bonded photonic EAM tensor core. The blue box indicates the region shown in **a**. **c**, Edge detection of digit ‘0’ using Sobel *G*_*x*_ and Sobel *G*_*y*_ filters. **d**,**e**, Convolution accuracy of using a partially coherent system without average (**d**) and with four-point average (**e**). A total of 100,000 pairs of expected and measured results are compared in both **d** and **e**. The insets show the Gaussian distribution of normalized errors. **f**, CNN architecture. **g**, Comparison of CNN classification results. Source Data

The applicability of partially coherent systems extends beyond the photonic memory tensor core described above, which is operated at a modest modulation speed of 1 kHz for specialized applications such as gait-signal classification. The versatility of the general approach caters to any photonic weighting device using amplitude modulation and is proficient at performing high-speed convolutional processing for diverse AI tasks. This is demonstrated through a high-speed 9 × 3 silicon photonic tensor core using EAMs, equipped with an integrated input EAM array and output photodetector array (Fig. 5a,b). The chip is fabricated using IMEC’s iSiPP50G silicon photonics platform, which provides the active components at a higher integration level. Hereafter we refer to this system as a photonic EAM tensor core. Using a field-programmable gate array (FPGA)-controlled electro-optic interface to the photonic EAM tensor core, we perform convolutional processing on the MNIST handwritten digits dataset at a data-loading rate of 2 gigasamples per second (GSa s^−1^) in each channel, using 8.0-nm-bandwidth partially coherent light. This 2 GSa s^−1^ data-loading rate brings the total system processing speed to 0.108 TOPS considering the size of the photonic tensor core, and an estimated energy efficiency of 1 TOPS W^−1^ (Supplementary Text 4). Supplementary Fig. 14 illustrates the configuration and data flow of the entire system, which operates analogously to the 3 × 3 photonic memory tensor core described above. Using the digit ‘0’ from the MNIST dataset as an example (Fig. 5c), the 2 GSa s^−1^ partially coherent system effectively extracts edges using Sobel *G*_*x*_ and Sobel *G*_*y*_ filters, albeit with increased background noise. As the noise originates from the stochastic properties of the ASE light source, it can be mitigated by averaging further convolutions per sample. Quantitatively, the normalized standard deviation of the error is 0.094 without averaging (Fig. 5d), which is reduced to 0.049 by four-point averaging (Fig. 5e). When the convolution results are used as input to a CNN for classification (Fig. 5f), accuracies of 92.4% without averaging and 93.9% with four-point average are achieved, closely aligning with the theoretical accuracy of 95.0% attained from CPU-implemented convolutions. The corresponding confusion maps and evolution of loss and accuracy with respect to increasing epochs are presented in Supplementary Figs. 15 and 16. Furthermore, the convolutional processing on the MNIST fashion products dataset, executed using the same system, reveals similar performance trends, detailed in Supplementary Text 5. We wish to note that the 2 GSa s^−1^ data-loading rate is limited by the digital-to-analogue converters (DACs) of the FPGA and not the photonic chip. The partially coherent light can provide a data-loading rate of at least 30 GSa s^−1^ (Supplementary Fig. 21), which brings the total system processing speed to 1.62 TOPS per optical carrier. Furthermore, using an ASE optical bandwidth of 40 nm for ten optical carriers (4 nm optical bandwidth per optical carrier), the partially coherent system is expected to reach 16.2 TOPS system processing speed.

## Discussion and conclusion

We have demonstrated that decreasing optical coherence can lead to enhanced performance in photonic computing systems, challenging the conventional wisdom that a higher degree of coherence is always advantageous. By decreasing the degree of coherence, we effectively exploit the optical bandwidth to boost parallelism without substantially degrading convolution accuracy. Specifically, reducing the coherence of the input light sources enables the same wavelength to be distributed across all input channels of a photonic tensor core. This also implies better scalability of photonic tensor cores, as the input optical bandwidth does not scale with the input vector dimension and thus is not limited by the spectral window of photonic components. In a system with an *N* × *N* photonic tensor core and *P* × *N* available wavelengths, partially coherent light facilitates *P* × *N* parallel convolutional processing operations, whereas coherent light facilitates *P* parallel operations. The limitations of partially coherent systems are related to the intrinsically reduced SNR, which is attributed to the stochastic properties of ASE. Considering these advantages and limitations, a quantitative comparison between coherent and partially coherent systems is shown in Supplementary Text 6. This comparison suggests that, although coherent systems exhibit advantages at the small scale by delivering high SNRs and modest parallelism, partially coherent systems surpass them at larger scales by offering enhanced parallelism and comparable SNRs. Furthermore, the SNR of partially coherent light may be improved by the substitution of EDFA ASE with broadband SLEDs^45^ and further optimized by coupling with saturated semiconductor optical amplifiers^46^. SLEDs have high spatial coherence with the benefit of easier coupling to the waveguide, moderate optical bandwidth (a few nanometres to tens of nanometres) for partial coherence control and favourable optical power^39,40^. We also note that the long delay lines required in large partially coherent systems are challenging to implement. The solutions to address this long delay line issue are discussed in detail in Supplementary Text 7. Assuming that we require the loss of the longest delay line to be below 3 dB and we use only one ASE source with an optical bandwidth of 4 nm, the system can support approximately a maximum of 59 input channels on a silicon nitride-on-silicon platform with a propagation loss of 0.4 dB cm^−1^ (refs. ^47,48^). To realize larger partially coherent systems, we can use an array of independent ASE sources working at the same wavelength, with each ASE source driving a few tens of input waveguide channels. These independent ASE sources are uncorrelated, eliminating the need for longer delay lines to overcome the coherence length of a single source. Using numerous ASE sources is still advantageous compared with using numerous lasers because each laser can only drive one input channel and independent lasers should still use different wavelengths to avoid undesired interference^49,50^.

As a proof of concept, we used partially coherent light in a system featuring a 3 × 3 photonic memory tensor core to demonstrate the parallel convolution of two gait signals from patients with Parkinson’s disease. These convolution results were subsequently used for CNN classification. Comparable convolutional processing and CNN classification accuracies were achieved as compared with using coherent light, while conserving four optical bands. To illustrate the broad applicability of partially coherent systems for high-speed convolutional processing in more complex AI tasks, we demonstrated 0.108 TOPS convolutional processing on MNIST handwritten digits dataset using a 9 × 3 photonic EAM tensor core with integrated input modulators and output photodetectors. The CNN classification accuracy reaches 92.4%, slightly below the theoretical accuracy of 95.0%, yet improvable to 93.9% through four-point average. A comparative analysis with other prevailing state-of-the-art photonic computing systems is provided in Supplementary Text 8. Our partially coherent system uniquely features phase insensitivity throughout the whole system. This technological shift away from coherent light considerably alleviates system requirements by circumventing stringent light-source specifications and eliminating the need for numerous precise phase controls, MRR controls and thermal management. Our findings suggest that EDFA ASE, SLEDs or other simple light sources can be used to bolster photonic computing performance rather than diminish it. This insight has the potential to revolutionize photonic computing systems as they evolve to accommodate increasingly complex computational tasks and continue to scale up to large *N* and *P* values.

## Methods

### Device fabrication

#### MZI array

The fabrication started from a silicon-on-insulator wafer (SOITEC) with a 220-nm silicon (Si) device layer and a 2-µm buried oxide layer. A 200-nm-thick positive e-beam resist (CSAR 62) was spin-coated on a diced 1 cm × 1 cm silicon-on-insulator chip, followed by 3 min pre-bake at 150 °C. The e-beam resist was patterned by e-beam lithography (EBL; JEOL JBX-5500 50 kV) and developed in AR 600-546 for 30 s, MIBK for 15 s and IPA for 15 s in sequence. The waveguide patterns were transferred to the Si device layer (etch depth = 110 nm) by reactive ion etching (Oxford Instruments PlasmaPro) with SF_6_ and CHF_3_ gases, followed by O_2_ plasma cleaning of CSAR. A 1-µm-thick silicon dioxide (SiO_2_) was deposited by plasma-enhanced chemical vapour deposition (Oxford Instruments PlasmaPro) as the upper cladding layer to isolate waveguides from thermo-optic phase shifters. Next, a 2-µm-thick double-layer PMMA (PMMA 495 A8 and PMMA 950 A4) was spin-coated on the chip, followed by EBL patterning and development in MIBK:IPA = 1:3 for 1 min to define the heater patterns. A 200-nm-thick NiCr layer was sputtered using a magnetron sputtering system (physical vapour deposition, AJA International), followed by PMMA lift-off to form NiCr heaters. Gold pads of 100 nm thickness were fabricated using a similar process as NiCr heater fabrication, but with e-beam evaporation (Plassys MEB550S). A 3–5-nm Cr layer was deposited before gold deposition to serve as an adhesion layer. The optical image of the fabricated MZI array is shown in Supplementary Fig. 1.

#### Photonic memory crossbar array

The Si photonic circuit was fabricated using the foundry multi-project wafer service provided by CORNERSTONE. The detailed specifications of CORNERSTONE standard waveguide components can be found at https://cornerstone.sotonfab.co.uk/. The fabricated Si photonic circuit has a 1-µm-thick SiO_2_ upper cladding. SiO_2_ windows were patterned by EBL and opened by hydrogen fluoride for the following deposition of the Ge_2_Sb_2_Te_5_ (GST)/indium tin oxide (ITO) stack. Next, GST/ITO stack windows were opened by the above-mentioned PMMA process. A 10-nm-thick/10-nm-thick GST/ITO stack was deposited on the waveguide using a magnetron sputtering system (physical vapour deposition, AJA International). The GST and ITO targets were respectively sputtered at 30 W RF power with 3 sccm Ar flow and 40 W RF power with 3 sccm Ar flow at a base pressure of 10^−7^ torr. The stack was then lifted off in acetone for 180 min at 50 °C. Next, the thermo-optic phase shifters were fabricated using the method described for the MZI array. Finally, the chip was annealed on a hotplate for 5 min at 250 °C to fully crystallize the GST. The fabricated photonic memory crossbar array is shown in Fig. 3a.

#### Photonic EAM tensor core

The photonic EAM tensor core was fabricated using the foundry multi-project wafer service provided by IMEC: iSiPP50G, with details at https://www.imeciclink.com/en/asic-fabrication/si. This platform provides the monolithic integration of passive waveguide circuits, integrated EAMs and integrated photodetectors used in the photonic EAM tensor core.

### Measurement setup

#### Coherence property measurement

The coherent light was generated by a tunable coherent laser (Santec, TSL-550) operating at 1,550 nm. The 0.8-nm-bandwidth C34 partially coherent light was generated by filtering the ASE from an EDFA (Pritel FA-33) with a passive DEMUX module (Gezhi, DWDM-100G-DEMUX) operating at channel C34 of the ITU grid. The 2.0, 4.0, 8.0 and 16.0-nm-bandwidth partially coherent light sources were generated by filtering the same ASE with an optical tunable band-pass filter (Santec, OTF-350) operating at a centre wavelength of 1,550 nm. The spectra were measured by an optical spectrum analyser (Anritsu, MS9710C). For eye diagrams, light was modulated by a pulse generator (Agilent, 8133A) through an electro-optic modulator (Lucent 2623N) and received by a photodetector (Newport New Focus 1611) connected to an oscilloscope (Tektronix, TDS7404B).

#### System setup for parallel convolutional processing

The experimental setup for parallel convolutional processing on two gait signals is shown in Fig. 4a. The photonic memory crossbar array has three input channels and three output channels, representing a *d*_3×3_ matrix consisting of three *d*_1×3_ kernels. The input light was switchable between an EDFA (Pritel FA-33) and a tunable pump laser (Santec, TSL-550) using an optical switch (Gezhi GZ-12C-1×2-SM). The phase-change-material photonic memory in each cell of the photonic memory crossbar array was first set to the desired weight to correctly define kernels. The tunable pump laser was used in phase-change-material weight setting. The amplified pump light passed through a DEMUX module (Gezhi, DWDM-100G-DEMUX) so that different wavelengths were routed to different input channels (*λ*_1_ = 1,550.12 nm to Ch 1, *λ*_2_ = 1,550.92 nm to Ch 2 and *λ*_3_ = 1,551.72 nm to Ch 3). After setting all phase-change-material weights, parallel convolution was performed using the ASE from the EDFA. The DEMUX module was used to separate two wavelengths with a spacing of 0.8 nm to two different channels (*λ*_1_ = 1,550.12 nm and *λ*_2_ = 1,550.92 nm). Each wavelength was split into three channels by an optical splitter (FS PLC splitter). The three channels serve as the input light to the three respective input waveguide channels of the photonic memory tensor core. Adjacent channels have a 1-m path difference, using a further 1-m-long fibre to eliminate the coherence among all three input light sources. The gait-signal data were loaded into each channel using a variable optical attenuator (VOA; Thorlabs V1550A). The VOAs were driven by a digital signal processor (DSP; NI USB-6259). The polarization of output light from the VOA was controlled by a polarization controller (Thorlabs FPC032). Different wavelengths carrying the gait signal at the same time index from different patients were then grouped by a MUX array (Gezhi, DWDM-100G-MUX) to form three inputs to the respective input channels of the photonic memory tensor core. Convolutions were performed naturally as light propagated through the photonic memory crossbar array. Each output channel of the photonic memory tensor core contained both wavelengths *λ*_1_ and *λ*_2_. The two wavelengths were demultiplexed to obtain the outputs and detected by a photodetector array (Newport New Focus 2011) and finally read out from the DSP.

#### System setup for high-speed convolutional processing

The experimental setup for high-speed convolutional processing on the MNIST datasets is shown in Supplementary Fig. 13. The whole system operating at 2 GSa s^−1^ was controlled by a FPGA evaluation board (Xilinx, Zynq UltraScale+ RFSoC ZCU216) with a processing system unit, a programmable logic unit, 16 DACs and 16 analogue-to-digital controllers. The optical input was the 8.0-nm-bandwidth partially coherent light equally split into nine input grating couplers. The MNIST data were read by the processing system unit, stored in its DDR4 memory and accessed by the programmable logic unit to output at nine analogue-to-digital controllers that modulated optical signals through the input EAM array. The weights on the photonic EAM crossbar array were set by a low-speed DSP. The three convolutional processing outputs were received by the integrated photodetector array connected to three transimpedance amplifiers and analogue-to-digital controllers, routed back to the processing system unit and stored in DDR4 memory.

### Mapping non-negative transmission to negative convolution results

The input gait signals and image data presented in this work are non-negative, that is, *x* ∈ [0, 1]. The kernels involve negative values, that is, *w* ∈ [−1, 1]. The measurable outputs from the photonic system are non-negative as a result of them being physical quantities. We need to map these non-negative outputs to convolution results in the range [−1, 1]. This is done by the following steps:

- We normalize every gait signal or image data to [0, 1] using software and load these normalized data to the photonic tensor core using modulators.
- We represent the input data *x* using the output power of the modulator by setting *P* = *x*(*P*_max_ − *P*_min_) + *P*_min_, in which *P*_max_ and *P*_min_ are the maximum and minimum outputs from the modulator, respectively.
- We represent the weight *w* using the transmission level of the phase-change material or the EAM by setting $T=w\left(\frac{{T}_{\max }-{T}_{\min }}{2}\right)+\frac{{T}_{\max }+{T}_{\min }}{2}$, in which *T*_max_ and *T*_min_ are the maximum and minimum transmission levels of the weight-setting device, respectively.
- We set the input vector **x** to the target input data and set the kernel **w** to the target weights. The measured output is:
  1 $${\sum }_{i}{P}_{i}\times {T}_{i}={\sum }_{i}\left[({P}_{\max }-{P}_{\min })\left(\frac{{T}_{\max }-{T}_{\min }}{2}\right){x}_{i}{w}_{i}+({P}_{\max }-{P}_{\min })\frac{{T}_{\max }+{T}_{\min }}{2}{x}_{i}+{P}_{\min }\left(\frac{{T}_{\max }-{T}_{\min }}{2}\right){w}_{i}+{P}_{\min }\frac{{T}_{\max }+{T}_{\min }}{2}\right]$$
  Step (d) should be performed for every input vector **x**.
- We set all *x* = 0 and all *w* = 0. Thus all *P* = *P*_min_ and all $T=\frac{{T}_{\max }+{T}_{\min }}{2}$. The measured output is:
  2 $${\sum }_{i}{P}_{\min }\frac{{T}_{\max }+{T}_{\min }}{2}$$
  Step (e) only needs to be performed once for the whole system.
- We set all **x** = 0 and set **w** to the target weights. Thus all *P* = *P*_min_ and ${T}_{i}={w}_{i}\left(\frac{{T}_{\max }-{T}_{\min }}{2}\right)+\frac{{T}_{\max }+{T}_{\min }}{2}$. The measured output is:
  3 $${\sum }_{i}\left[{P}_{\min }\left(\frac{{T}_{\max }-{T}_{\min }}{2}\right){w}_{i}+{P}_{\min }\frac{{T}_{\max }+{T}_{\min }}{2}\right]$$
  Step (f) needs to be performed once for each kernel.
- We set **x** to the target input data and set all **w** = 0. Thus *P*_*i*_ = *x*_*i*_(*P*_max_ − *P*_min_) + *P*_min_ and all $T=\frac{{T}_{\max }+{T}_{\min }}{2}$. The measured output is:
  4 $${\sum }_{i}\left[\left({P}_{\max }-{P}_{\min }\right)\frac{{T}_{\max }+{T}_{\min }}{2}{x}_{i}+{P}_{\min }\frac{{T}_{\max }+{T}_{\min }}{2}\right]$$
  Step (g) should be performed for every input vector **x**.
- We perform post-processing on a computer using the measured output from steps (d)–(g) as:
  5 $${\rm{Result}}=\left(1\right)-\left(3\right)-\left(4\right)+\left(2\right)=\left({P}_{\max }-{P}_{\min }\right)\left(\frac{{T}_{\max }-{T}_{\min }}{2}\right){\sum }_{i}{x}_{i}{w}_{i}$$
- We normalize the results to [−1, 1] using software because all results share the same factor of ${(P}_{\max }-{P}_{\min })(\frac{{T}_{\max }-{T}_{\min }}{2})$ and *x* ∈ [0, 1] and *w* ∈ [−1, 1].

We can see that the hardware computation is doubled using this mapping approach, yet this mapping approach can be implemented without doubling by hardware implementation involving a balanced photodetection scheme (Supplementary Text 2).

### Generation, convolution and output of gait signals

The properties of the original gait-signal data collected by force sensors (Ultraflex Computer Dyno Graphy, Infotronic) are described in the next section ‘CNN model; Gait-signal dataset’.

For parallel convolution of the middle three time-domain data of two gait signals, the input matrix is a *d*_3×2_ matrix: $$X=\left[\begin{array}{cc}{x}_{11} & {x}_{12}\\ {x}_{21} & {x}_{22}\\ {x}_{31} & {x}_{32}\end{array}\right]$$. The *j*th column of *X* contains the middle three time-domain data of the *j*th gait signal (Fig. 4). The *i*th row of *X* contains the *i*th time-domain data of two gait signals. A DSP drove VOAs to load gait signals into the optical domain. The photonic memory tensor core was then effectively performing:

$$\begin{array}{c}{Y=W\times X=\left[\begin{array}{ccc}{w}_{11} & {w}_{12} & {w}_{13}\\ {w}_{21} & {w}_{22} & {w}_{23}\\ {w}_{31} & {w}_{32} & {w}_{33}\end{array}\right]}^{{\rm{T}}}\left[\begin{array}{cc}{x}_{11} & {x}_{12}\\ {x}_{21} & {x}_{22}\\ {x}_{31} & {x}_{32}\end{array}\right]\\ \,\,\,\,=\,\left[\begin{array}{cc}\mathop{\sum }\limits_{n=1}^{3}{{w}_{n1}x}_{n1} & \mathop{\sum }\limits_{n=1}^{3}{{w}_{n1}x}_{n2}\\ \mathop{\sum }\limits_{n=1}^{3}{{w}_{n2}x}_{n1} & \mathop{\sum }\limits_{n=1}^{3}{{w}_{n2}x}_{n2}\\ \mathop{\sum }\limits_{n=1}^{3}{{w}_{n3}x}_{n1} & \mathop{\sum }\limits_{n=1}^{3}{{w}_{n3}x}_{n3}\end{array}\right]=\left[\begin{array}{cc}{y}_{11} & {y}_{12}\\ {y}_{21} & {y}_{22}\\ {y}_{31} & {y}_{32}\end{array}\right]\end{array}$$

in which ${y}_{{ij}}={\sum }_{n=1}^{3}{{w}_{{ni}}x}_{{nj}}$ represents the convolution result of the middle three time-domain data of the *j*th gait signal using the *i*th kernel. Each row of *Y* was output from the respective photonic memory tensor core output channel.

### CNN model

#### Gait-signal dataset

Gait signals from ten patients with Parkinson’s disease were taken from the ‘Gait in Parkinson’s Disease’ database in PhysioNet^51,52^. This database includes the vertical ground reaction force records of individuals as they walked at their usual, self-selected pace for approximately 2 min on level ground. The corresponding clinical information of ten patients is provided in Supplementary Table 1. Fifty gait pulses were extracted from each patient, leading to a total of 500 gait pulses. Each pulse has a 1.2-s duration. The original electrocardiogram signals have a 0.01-s time resolution. Gait pulses were extracted with a time interval of 0.04 s (that is, one out of every four original data), leading to 31 data in the extracted gait pulses. The 0.04-s time interval was carefully chosen to minimize the extracted dataset while maintaining the key features from the original gait pulses. Eighty per cent of pulses were used for training and 20% were used for testing, that is, a total of 400 pulses for training and 100 pulses for testing.

#### MNIST dataset

The test dataset of MNIST handwritten digits and MNIST fashion products were respectively taken from https://git-disl.github.io/GTDLBench/datasets/mnist_datasets/ and https://developer.ibm.com/exchanges/data/all/fashion-mnist/. In both cases, the 10,000 test images were split into a training set with 8,000 images and a testing set with 2,000 images.

#### CNN architecture

The CNN architecture for the classification of the gaits dataset is shown in Fig. 4d. The input layer takes the gait signal, which is in the form of a *d*_31×1_ 1D array. The 1D array is passed to a convolution layer consisting of three *d*_1×3_ kernels. Convolution operations were implemented with a stride of 1 and ‘valid padding’, resulting in a *d*_3×(31-3+1)_ output. The output was activated by a rectified linear unit layer and flattened to a *d*_87×1_ vector. The flattened activated output was then fed to a fully connected layer with ten neurons. The output from the fully connected layer was converted to probabilities by a softmax layer. Finally, the classification result was obtained. The gait signals were classified into ten categories, representing ten patients with Parkinson’s disease. The convolution operations were implemented using the photonic memory tensor core. The convolution results were processed by the following CNN layers using the MATLAB R2021b Deep Learning Toolbox. Weights of the fully connected layer were trained by the Adam optimizer. A hundred epochs were used to reach the final CNN outcomes. The CNN architecture for the MNIST datasets is similar to that for the gaits dataset, as shown in Fig. 5d. We will only mention the key differences here. For the MNIST datasets, besides the trivial difference in layer dimensions, the images were convolved with ‘same padding’ implemented by the photonic EAM tensor core. We used 50 epochs to reach the final CNN outcomes.

## Online content

Any methods, additional references, Nature Portfolio reporting summaries, source data, extended data, supplementary information, acknowledgements, peer review information; details of author contributions and competing interests; and statements of data and code availability are available at 10.1038/s41586-024-07590-y.

## Supplementary information

Supplementary Information

## Source data

Source Data Fig. 2

Source Data Fig. 3

Source Data Fig. 4

Source Data Fig. 5

## Data Availability

The data that support the findings of this study are available from the corresponding author on request. The gait dataset analysed in this study is available from the open source ‘Gait in Parkinson’s Disease’ in PhysioNet at 10.13026/C24H3N. The MNIST handwritten digits dataset is available at https://git-disl.github.io/GTDLBench/datasets/mnist_datasets/. The MNIST fashion products dataset is available at https://developer.ibm.com/exchanges/data/all/fashion-mnist/. Source data are provided with this paper.

## Code availability

The code used in this work is available from the authors on request.
