# Supplementary material for: Partial coherence enhances parallelized photonic computing
Source: Nature. 2024 Jul 31;632(8023):55–62. doi: 10.1038/s41586-024-07590-y (PMC11291273; doi:10.1038/s41586-024-07590-y)
Supplement: Supplementary file 1 — Supplementary Information [file 41586_2024_7590_MOESM1_ESM.pdf]

nature portfolio

https://doi.org/10.1038/s41586-024-07590-y

Supplementary information

Partial coherence enhances parallelized photonic computing

In the format provided by the authors and unedited

Supplementary Materials for

Partial coherence enhances parallelized photonic computing

*Bowei Dong[1,2,6], Frank Brückerhoff-Plückelmann[3,6], Lennart Meyer[3], Jelle Dijkstra[3], Ivonne Bente[4], Daniel Wendland[4], Akhil Varri[4], Samarth Aggarwal[1], Nikolaos Farmakidis[1], Mengyun Wang[1], Guoce Yang[1], June Sang Lee[1], Yuhan He[1], Emmanuel Gooskens[5], Dim-Lee Kwong[2], Peter Bienstman[5], Wolfram H. P. Pernice[3,4], and Harish Bhaskaran[1]**

[1]Department of Materials, University of Oxford, Oxford, OX1 3PH, UK
[2]Institute Of Microelectronics, Agency for Science, Technology and Research (A*STAR), 138634, Singapore.
[3]Kirchhoff-Institute for Physics, Heidelberg University; Im Neuenheimer Feld 227, 69120 Heidelberg, Germany.
[4]Center for Nanotechnology, University of Münster; Heisenbergstr. 11, 48149 Münster, Germany.
[5]Photonics Research Group, Ghent University – imec, Technologiepark-Zwijnaarde 126, B-9052 Gent, Belgium.
[6]These authors contributed equally: B. Dong, F. Brückerhoff-Plückelmann

*Correspondence to: harish.bhaskaran@materials.ox.ac.uk

Table of Contents

1. **Design considerations** **4**
   Power splitter design for equal input power distribution across all PCM weights 4
   Directional coupler design for the same output contribution from different unit cells 5
2. **Mapping transmission levels to negative weights** **7**
3. **Photonic computing system using coherent light** **9**
4. **Power efficiency estimation** **15**
5. **MNIST fashion products dataset results** **20**
6. **Limitation of partially coherent approach and comparison with coherent approach** **25**
7. **Solutions to address the long delay line issue** **30**
8. **Comparison with state-of-the-art photonic computing systems** **32**
9. **Supplementary References** **36**

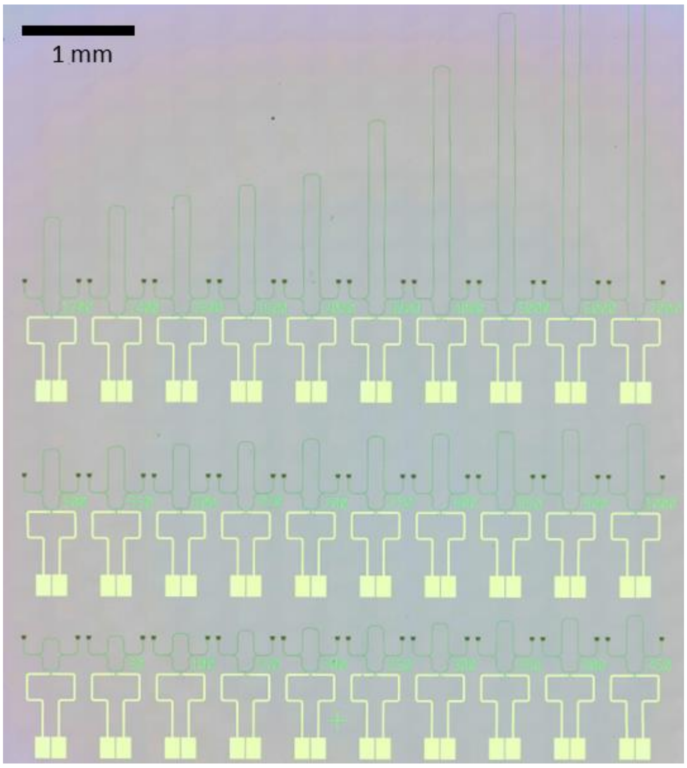

Figure.S1 Optical image of MZI array with increasing path length differences to determine the coherence lengths of coherent and partially coherent light sources.

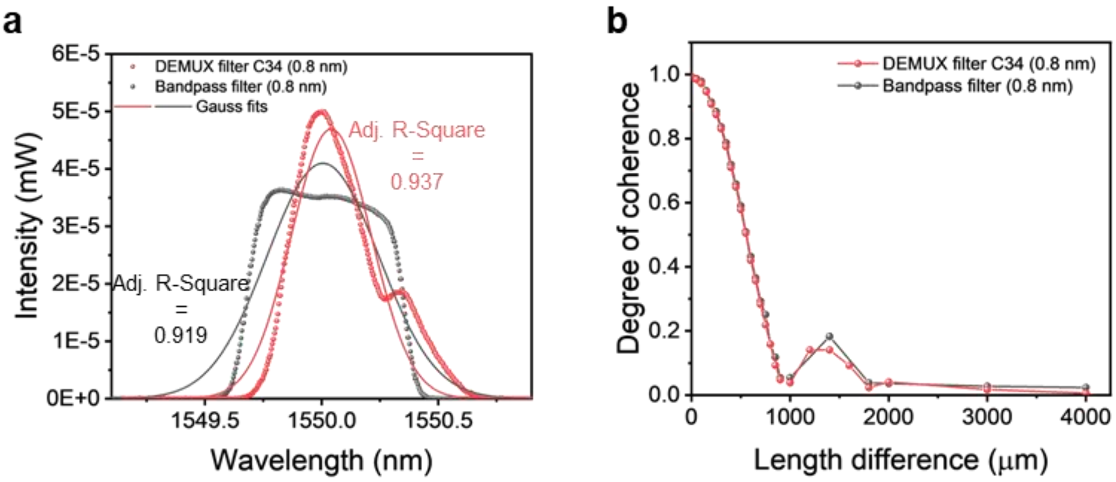

Figure.S2 Impact of non-Gaussian-shaped spectrum. a, Spectrum of a more-Gaussian-shaped partially coherent source and a less-Gaussian-shaped partially coherent source. **b,** Comparison of degree of coherence as a function of length difference.

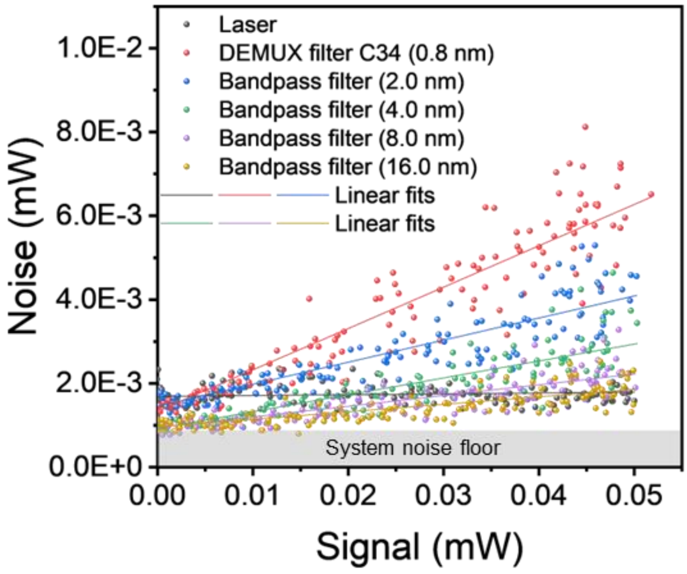

Figure.S3 Measured noise as a function of signal (intensity received at the photodetector).

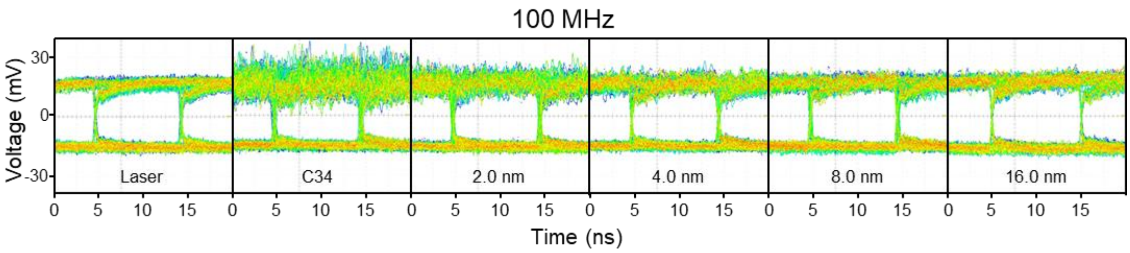

Figure.S4 Eye diagrams at 100 MHz.

1. Design considerations

Power splitter design for equal input power distribution across all PCM weights

PCM stands for phase-change material. Assume the photonic memory crossbar array has N input channels and M output channels. The equal power distribution is achieved by careful power splitter design (**Figure. S5a**). A power splitter is formed by a 1×2 multimode interferometer (MMI), a tunable Mach-Zehnder interferometer (MZI), and a 2×2 MMI in sequence. The input optical power to the mth cell of any row is $\frac{M-m+1}{m}$. The MZI determines the 2×2 MMI outputs by controlling the phases of two inputs, and is designed to transmit $\frac{M-m+1}{M} \times \frac{M-m}{M-m+1} = \frac{M-m}{M}$ power via the top MMI output to the next cell (m+1)th, and transmit $\frac{M-m+1}{M} \times \frac{1}{M-m+1} = \frac{1}{M}$ power via the bottom MMI output to PCM weight for multiplication. Hence, each PCM memory receives the identical optical power of $\frac{1}{M}$. The weighted output from PCM memory in row n and column m is $\frac{w_{nm}}{M}$.

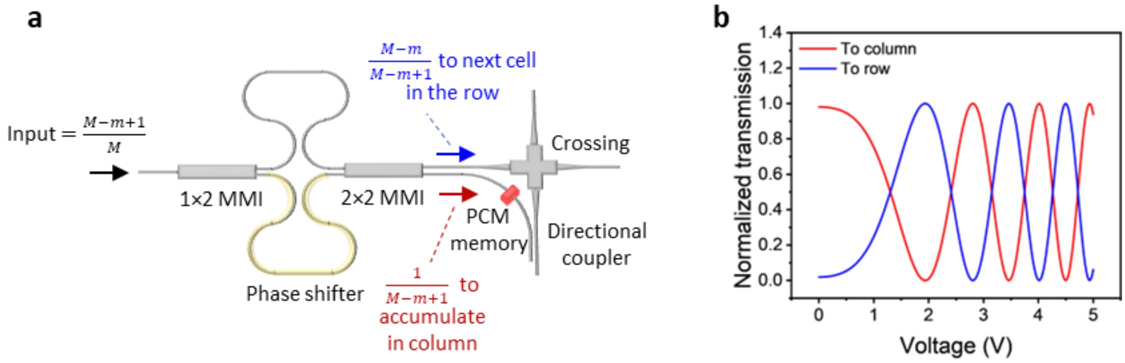

Figure.S5 Working principle of a single computation unit cell and relevant light routing performance. a, Structure of a single cell. **b,** Active tuning of power splitting ratio.

Directional coupler design for the same output contribution from different unit cells

The power splitters are equipped with active thermo-optic phase shifters to tune light routing across the photonic memory crossbar array. By supplying the proper voltage to the phase shifter, light can be completely routed to the next cell in a row for weight setting, or routed to the common bus waveguide in a row for accumulation (**Figure. S5b**). The tunable power splitters of photonic memory crossbar array were controlled by a digital signal processor (DSP, Analog Device DC2026) to ensure that all pump power was concentrated into PCM of the target cell. For example, to set w_{32} in **Fig. 4a**, λ_1 was used, VOA3 was on while VOA1 and VOA2 were off, so that the pump light was routed to Ch 3. $Cell_{31}$ was controlled to distribute all light into the top channel of its 2×2 MMI, and $Cell_{32}$ was controlled to distribute all light into the MMI bottom channel to efficiently set w_{32}. In this case, $Cell_{33}$ was idle.

Directional couplers (DC) are also carefully designed to ensure outputs from different unit cells have the same contribution. Since symmetric DCs are used to route weighted outputs from each cell into buses, the optical power in buses will partially couple back into cells. The coupling ratio of DCs in row n is designed to be $\frac{1}{n}$. Consequently, the optical power received at the output waveguide column m from row n is $\frac{w_{nm}}{M} \times \frac{1}{n} \times \left(1 - \frac{1}{n+1}\right) \times \left(1 - \frac{1}{n+2}\right) \times \cdots \times \left(1 - \frac{1}{N}\right) = \frac{w_{nm}}{N \times M}$, which is balanced across all cells except different weights.

-End of supplementary text 1

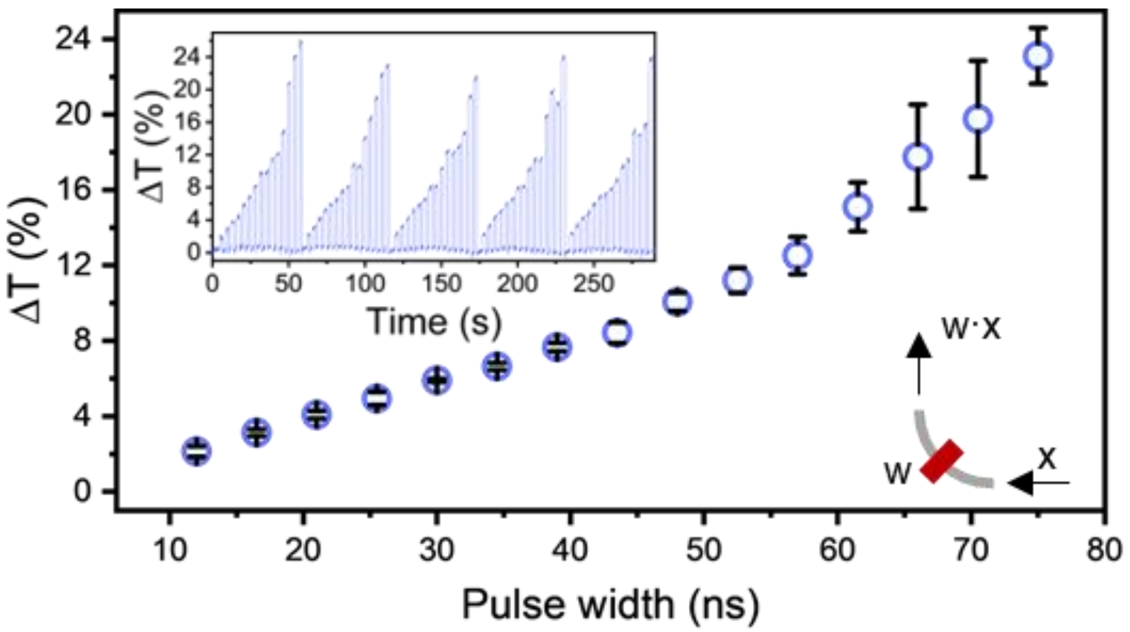

Figure.S6 Weight setting using a phase-change material photonic memory with 4-bit resolution. The error bars represent the standard deviation in transmission levels when the memory is set to a specific level.

2. Mapping transmission levels to negative weights

In our partially coherent photonic computing approach, the transmission levels of phase change material memory T are mapped to weights w in [-1,1] by defining $w = \frac{T - T_{ref}}{T_{ref}}$, where the reference transmission level is $T_{ref} = \frac{T_{max} + T_{min}}{2}$. This mapping approach can be implemented in hardware using the balanced detection scheme.

As shown in **Figure. S7a**, for every column implementing dot-product operation, we can add a reference column that stores all the reference transmission levels. The balanced photodetector will generate the mapped weight values that are allowed to be negative. The drawback of this scheme is the loss of half of the optical power.

Alternatively, instead of using a reference column for every dot-product operation, we can use only one reference column (**Figure. S7b**) and do the subtraction in software. This approach only leads to the loss of 1/(M+1) optical power, where M is the number of columns in the photonic tensor core. The running time of subtraction is thus $O(M)$, which is small compared to $O(M^2)$ MAC operations implemented by the photonic tensor core.

The need for such a balanced photodetection scheme is a consequence of the use of transmittance to represent weights. The transmittance, as a physical property, is non-negative. The third approach to address the non-negative weights issue, instead of changing the hardware architecture, involves the modification of neural network to adapt to the non-negative nature. The all-non-negative neural network has been investigated in Ref[1] and shows accuracy comparable to unconstrained neural networks for MNIST datasets, indicating all-non-negative neural network is possible,

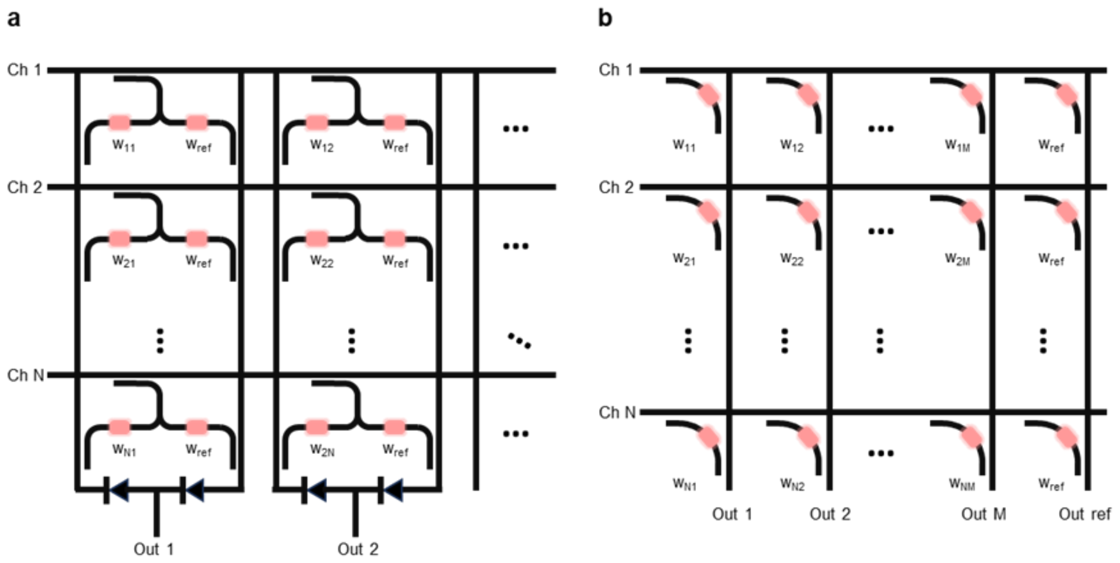

Figure.S7 Two possible hardware implementations for negative weights. a. Balanced photodetection scheme. **b.** Photonic tensor core with an additional reference column. The following subtractions are done in software.

-End of supplementary text 2

3. Photonic computing system using coherent light

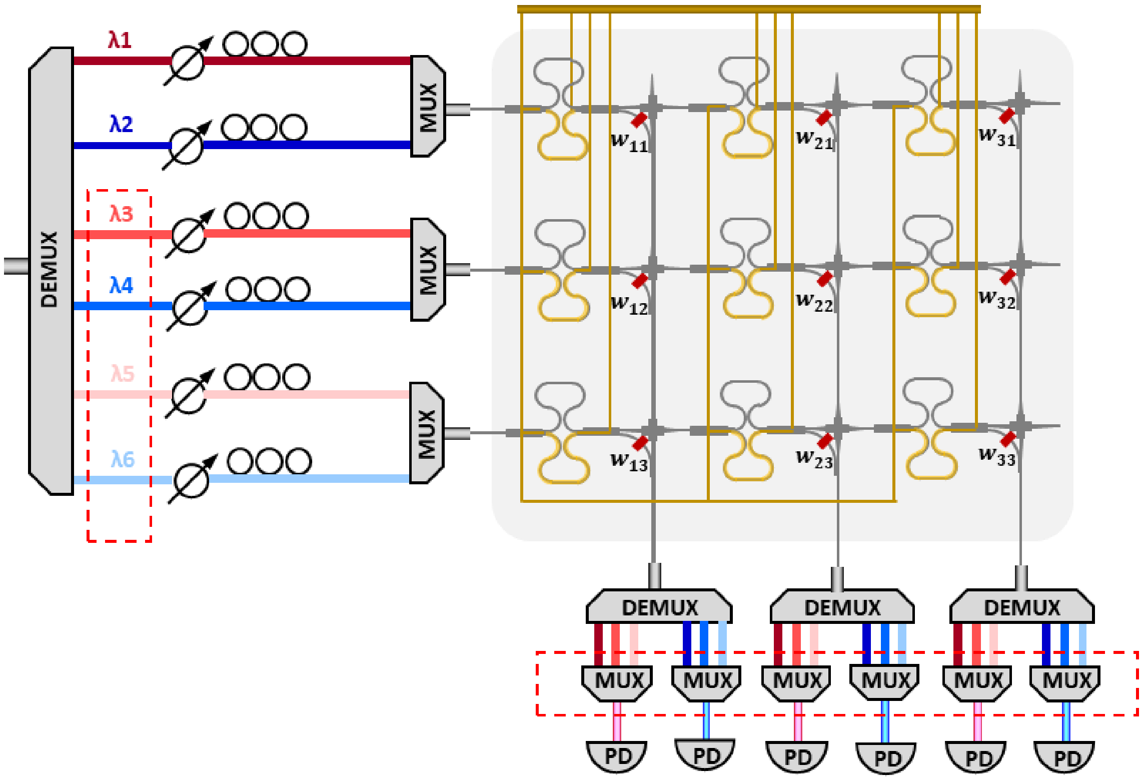

Figure.S8 Schematic of the computing system using the 3×3 photonic memory tensor core with a coherent light source. The extra photonic components required are highlighted in the red dashed boxes.

In the absence of optical delays, light from different input waveguides to the photonic memory tensor core are coherent. In order to prevent intensity fluctuation caused by phase fluctuation, different input waveguides should receive light at different wavelengths. Therefore, to implement the same convolutional processing operation in **Fig. 4a**, six wavelengths are required (**Figure.S8**). All three output channels contain light in all six wavelengths. The six wavelengths should be demultiplexed first, and then grouped again to represent the convolution results of individual gait signals (λ_1, λ_3, λ_5 form the output for patient 1, and λ_2, λ_4, λ_6 form the output for patient 2).

-End of supplementary text 3

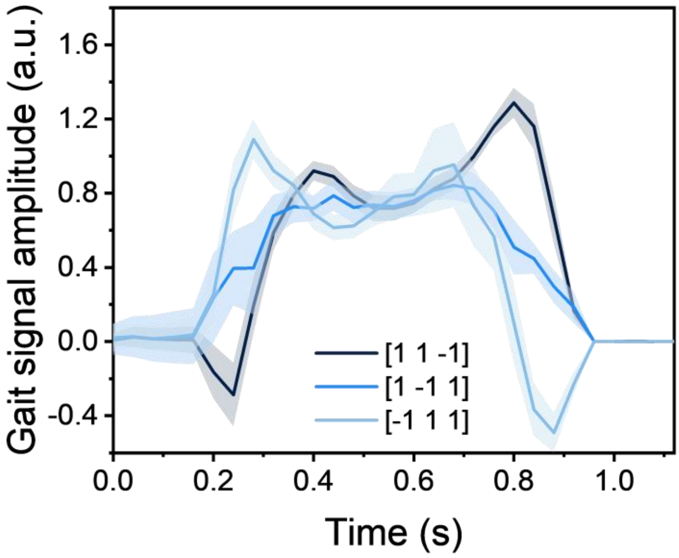

Figure.S9 Convolution results obtained using CPU. The error bands represent the standard deviation of convolution results from 50 gait signals generated by the same patient, showing the variation of gait signals from this patient.

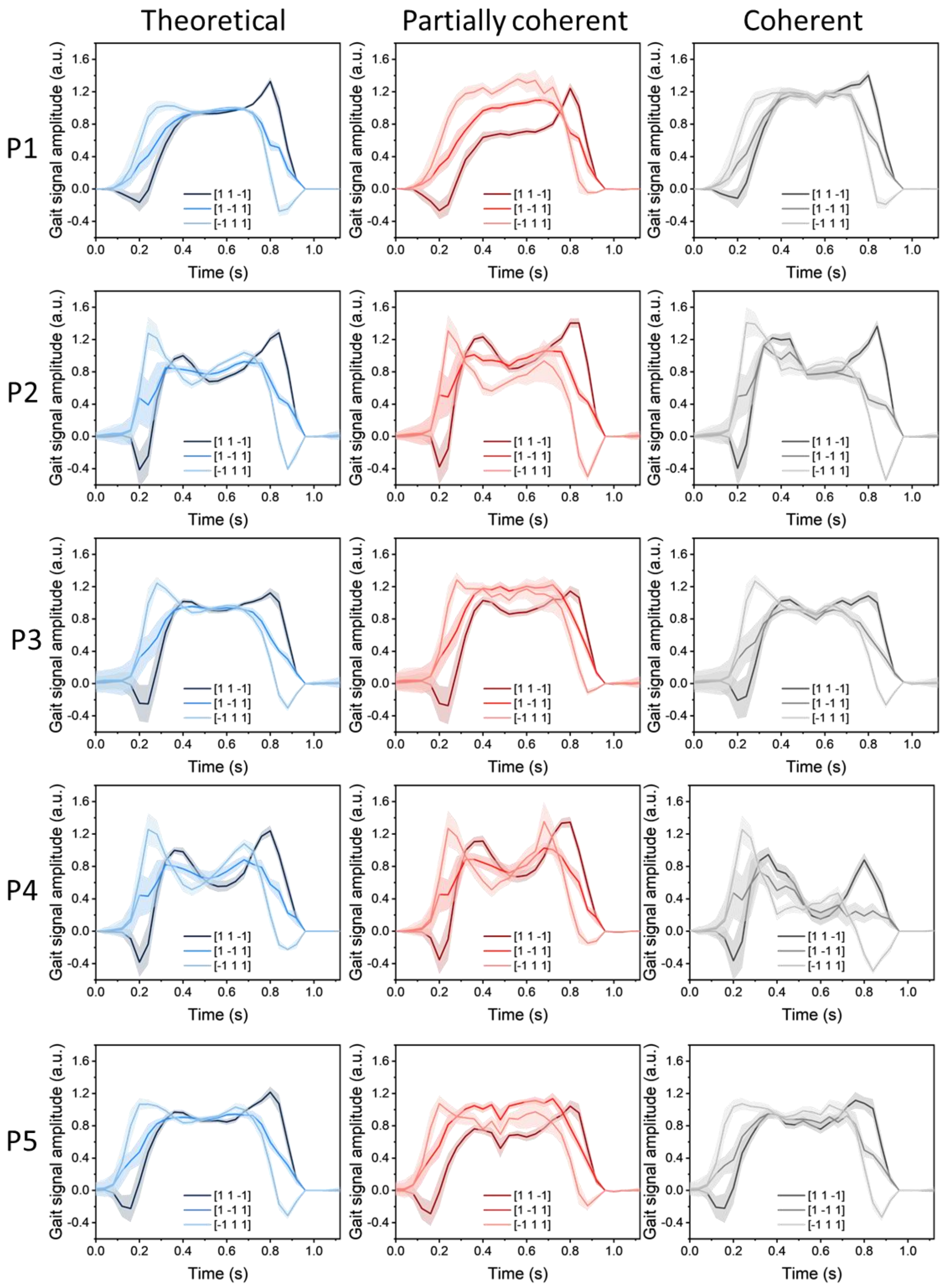

Figure.S10 Convolution results of gait signals from Parkinson's disease patients 1-5. The error bands represent the standard deviation of convolution results from 50 gait signals generated by the same patient, showing the variation of gait signals from this patient.

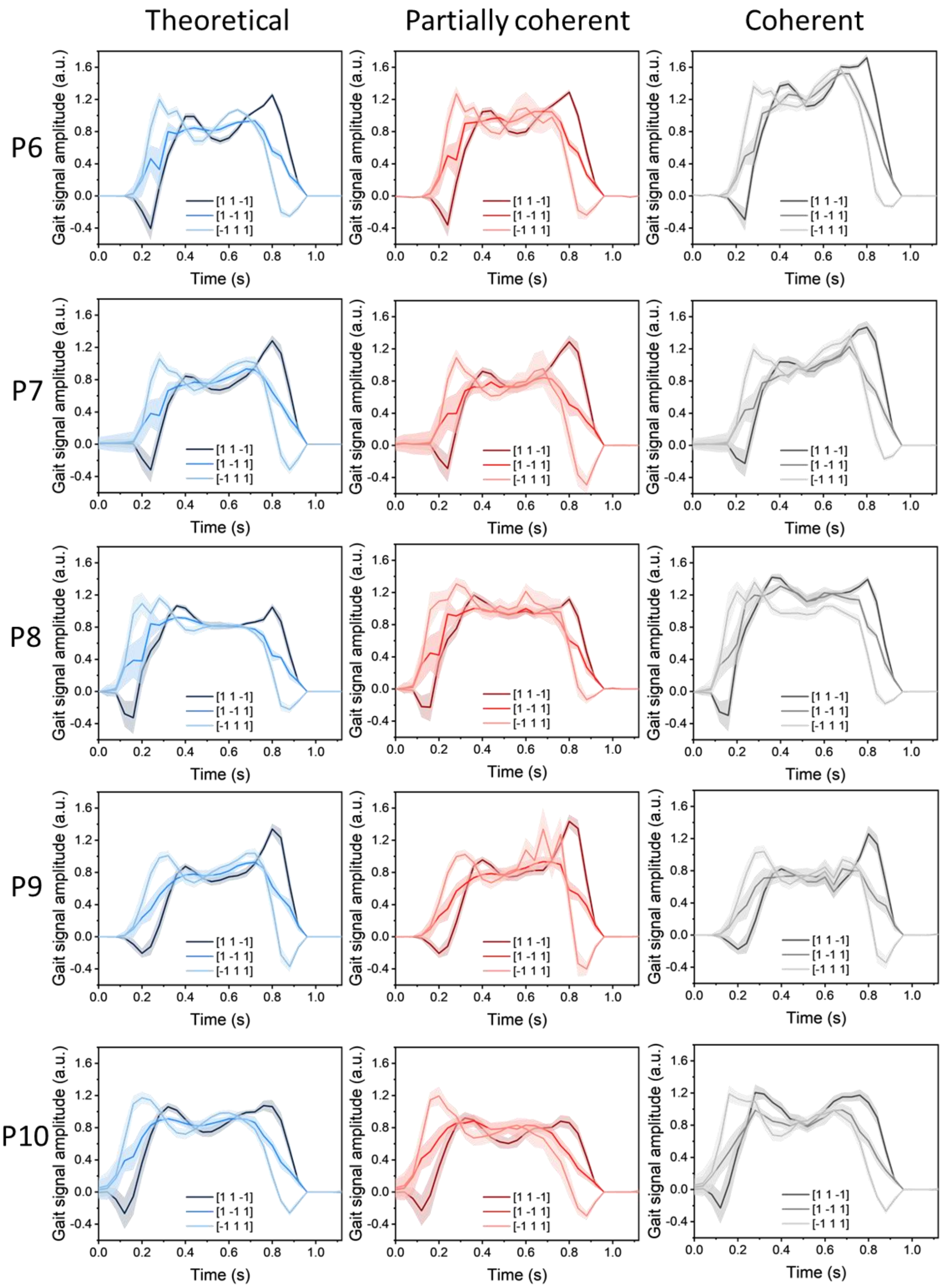

Figure.S11 Convolution results of gait signals from Parkinson's disease patients 6-10. The error bands represent the standard deviation of convolution results from 50 gait signals generated by the same patient, showing the variation of gait signals from this patient.

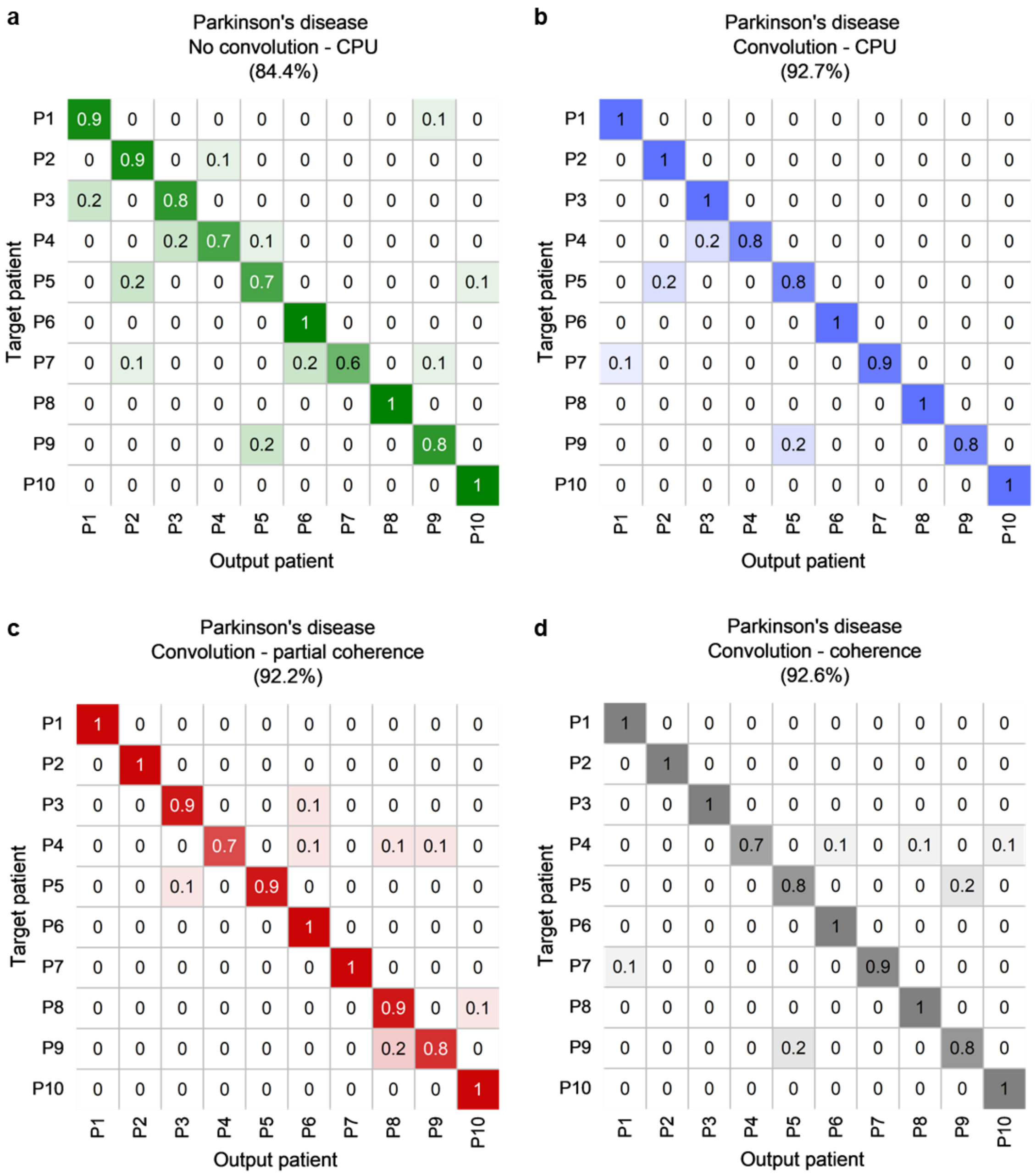

Figure.S12 Confusion maps of CNN classification results for identification of Parkinson's disease patients using their gaits. a, Without convolution. **b,** Convolution performed by CPU. **c,** Convolution performed by partially coherent system. **d,** Convolution performed by coherent system.

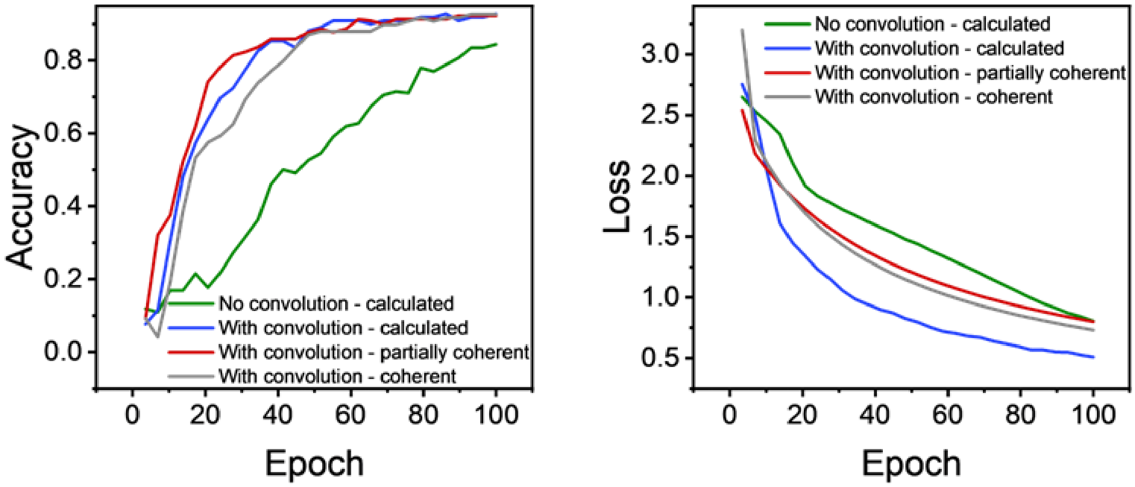

Figure.S13 Evolution of CNN loss and accuracy with increasing epochs in the identification of Parkinson's disease patients using their gaits.

4. Power efficiency estimation

The power of the partially coherent photonic computing system is attributed to 1) light source, 2) modulators for data loading, 3) electronics for data loading modulators, 4) weighting elements, 5) optical receivers, 6) ADCs.

Assuming a N×M photonic tensor core operating at f GSa/s, the expected power consumption will be:

1) P_{light} Light source. We use 0.2 mW at each input channel. Considering a wall-plug efficiency of 3.1% of integrated ASE source[2], the electrical power is 6.45 mW. The overall power consumption of light source will be:

$$6.45\times N\times 10^{-3}\ \text{W}$$

2) P_{mod} Modulators for data loading. The energy consumption of each IMEC EAM we use is 13.8 fJ/bit[3]. The overall power consumption of data loading modulators is:

$$13.8\times 10^{-15}\times f\times 10^{9}\times N = 13.8\times \text{f}\times \text{N}\times 10^{-6}\ \text{W}$$

3) $P_{mod\text{-}electronics}$. For data loading modulators, the electronics contain DAC, driver, and backends, which will consume 625 fJ/bit[4]. The overall power consumption of these electronics will be:

$$625\times 10^{-15}\times f\times 10^{9}\times N = 625\times \text{f}\times \text{N}\times 10^{-6}\ \text{W}$$

4) $P_{weigtht}$ Weighting elements. The use of phase-change material as non-volatile photonic memory will consume no power as the weight matrix presents fixed kernels.

5) P_{rec} Receivers. A receiver containing photodetector, TIA, and buffer will consume 170 fJ/bit[5]. The overall power consumption of receivers will be:

$$170\times 10^{-15}\times f\times 10^{9}\times M = 170\times f\times M\times 10^{-6}\ \text{W}$$

6) P_{ADC}. An ADC will consume 6.25 pJ/bit[6]. The overall power consumption of ADCs will be:

$$6250\times10^{-15}\times f\times10^{9}\times M = 6250\times f\times M\times10^{-6}\ \text{W}$$

The total throughput of the photonic tensor core will be:

$$2\times f\times10^{-3}\times N\times M\ \text{TOPS},$$

where the factor of 2 takes into account two operations in one multiply-accumulate operation.

The energy efficiency of the photonic tensor core is defined as:

$$\eta = Throughput\ /\ (P_{light}+P_{mod}+P_{mod\text{-}electronics}+P_{rec}+P_{ADC})\ \text{TOPS/W}$$

Considering the presented 9×3 photonic tensor core working at 2 GSa/s data loading rate, the energy efficiency will be:

$$0.108\ \text{TOPS} / (0.0580+0.0002+0.0112+0.0010+0.0375)\ \text{W} = 1\ \text{TOPS/W}$$

This 1 TOPS/W energy efficiency is equivalent to 1 TOPS/W in the latest Google TPUv4[7]. Energy efficiency of our system surpassing Google TPUv4 can be achieved at a larger tensor core size, because the throughput scales with N^2 while the energy consumption scales with N. A comparison of energy efficiency to other photonic computing systems is provided in Table. S2 in Supplementary Text 7.

-End of supplementary text 4

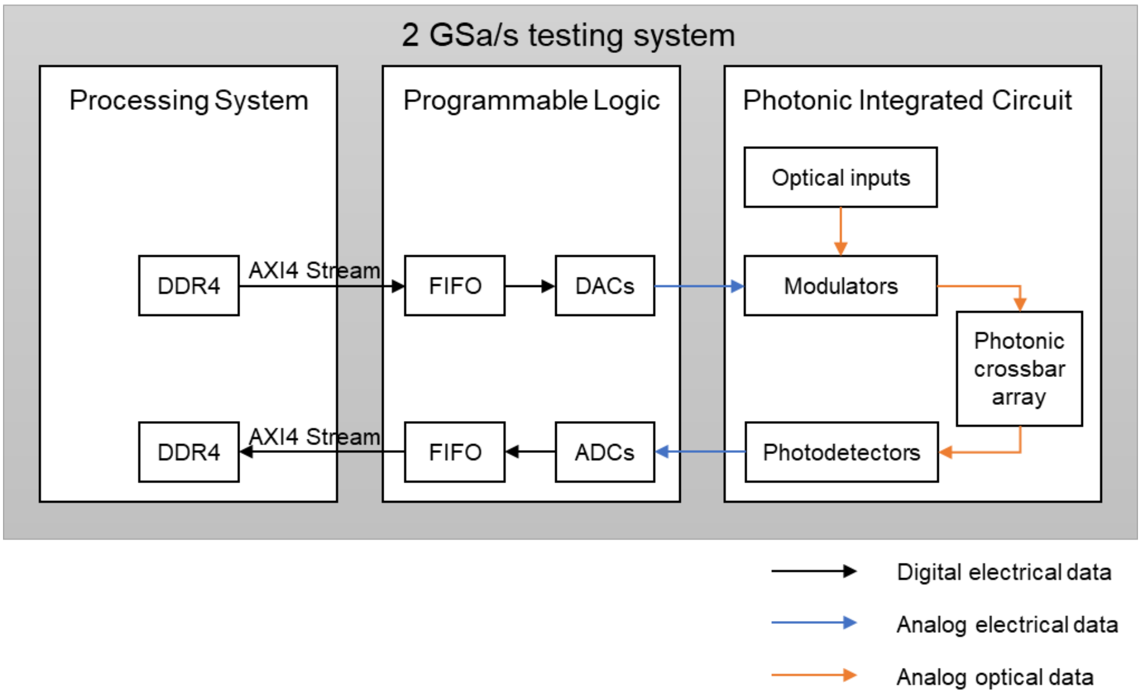

Figure.S14 Configuration and data flow of an FPGA-controlled photonic EAM tensor core operating at 2 GSa/s.

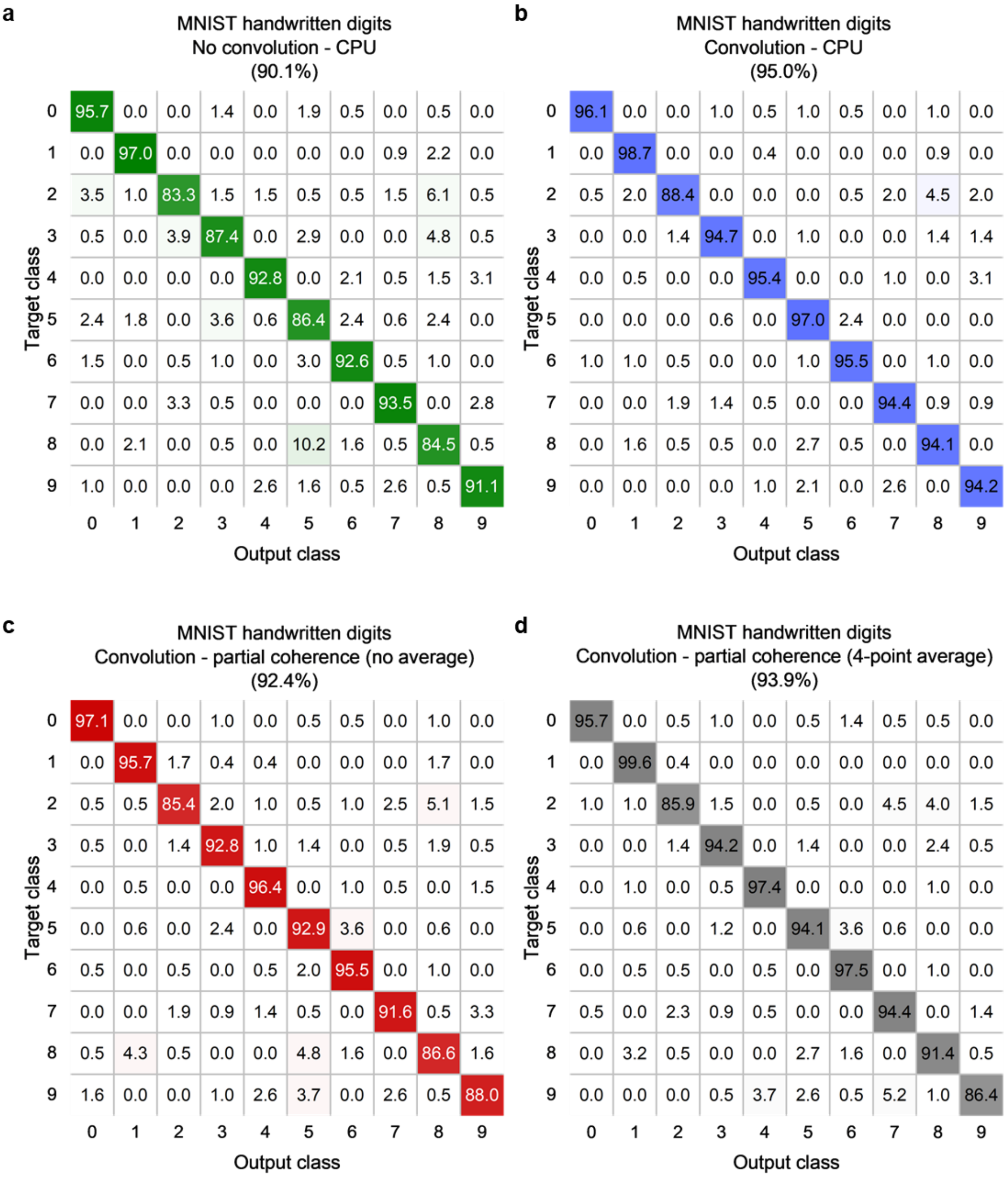

Figure.S15 Confusion maps of CNN classification results of MNIST handwritten dataset. a, Without convolution. **b,** Convolution performed by CPU. **c,** Convolution performed by partially coherent system without averaging. **d,** Convolution performed by partially coherent system with 4-point average.

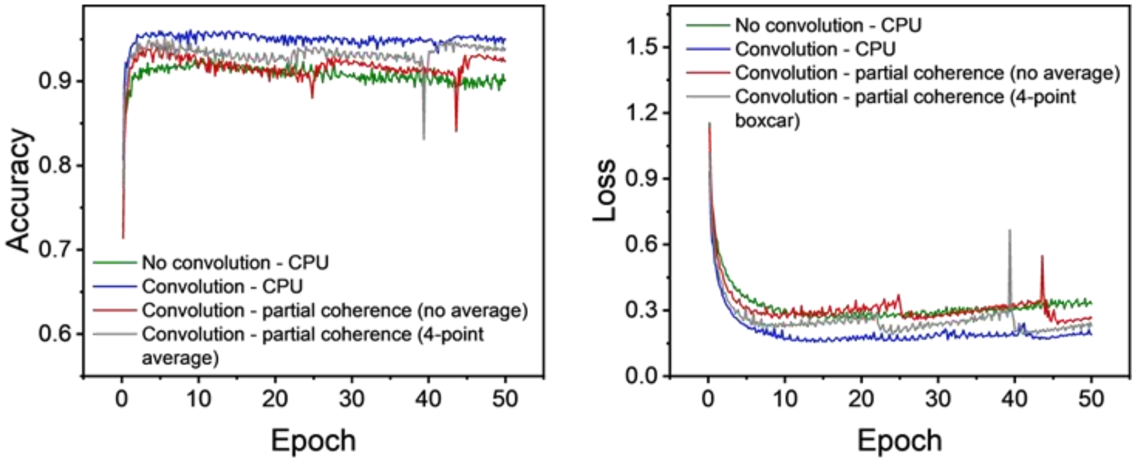

Figure.S16 Evolution of CNN loss and accuracy with increasing epochs in the classification of MNIST handwritten digits dataset.

5. MNIST fashion products dataset results

The results are qualitatively similar to the MNIST handwritten digits dataset, wherein edges are effectively extracted amidst certain background noise (**Figure. S17a**), and this noise is reduced by 4-point average (**Figure. S17b&c**). Nonetheless, quantitatively, the classification accuracy achieved in the MNIST fashion products dataset is lower compared to the handwritten digits dataset. As shown in **Figure. S17d**, using convolution results obtained by CPU, the classification accuracy is 82.8%. However, while the accuracy remains high at 80.2% with 4-point average, it declines to 74.6% without averaging. Associated confusion maps and evolution of loss and accuracy with respect to increasing epochs are presented in **Figure. S18** and **Figure. S19**. This discrepancy in accuracy can be explained by the different noise resilience between the MNIST handwritten digits dataset and MNIST fashion product dataset under the specific CNN configuration (**Figure. S20**), and could be improved by enhancing the SNR of partially coherent systems or adjusting the CNN configuration.

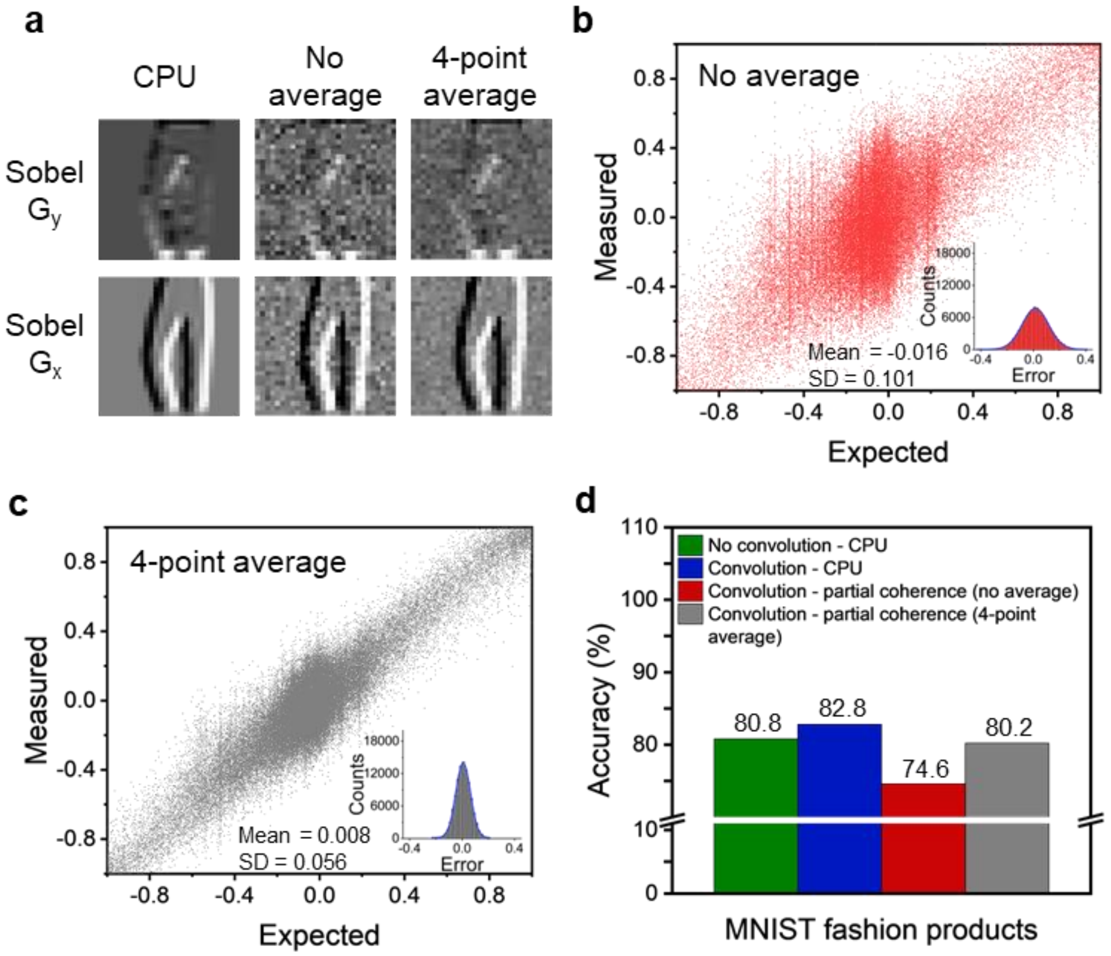

Figure.S17 Convolution results and CNN classification accuracy of MNIST fashion products dataset. a, An example of trouser edge detection. **b,** Convolution accuracy without averaging. **c,** Convolution accuracy with 4-point average. A total of 100,000 pairs of expected and measured results are compared in both b and c. The insets show the Gaussian distribution of normalized errors. **d,** Comparison of CNN classification results.

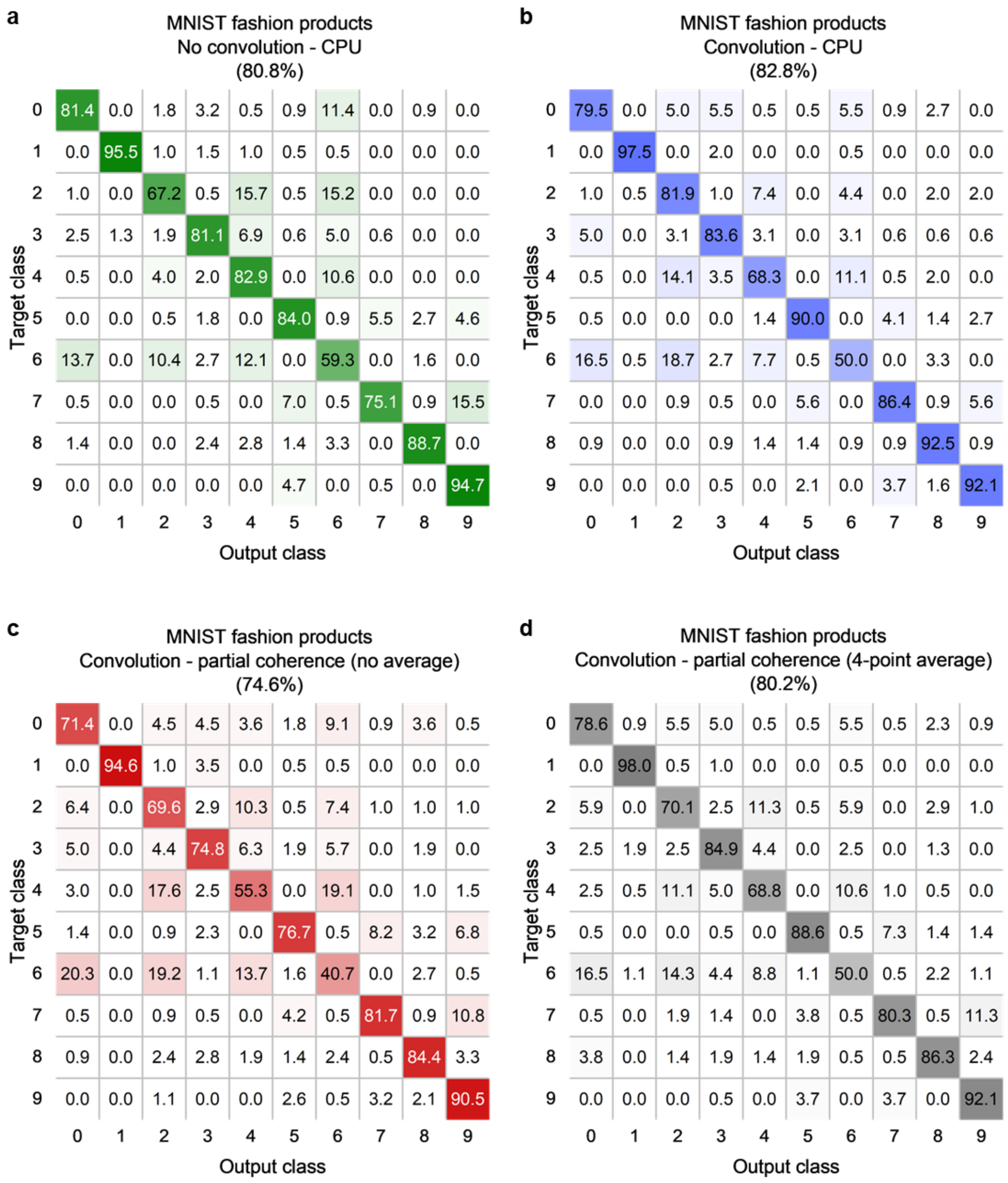

Figure.S18 Confusion maps of CNN classification results of MNIST fashion products dataset. a, Without convolution. **b,** Convolution performed by CPU. **c,** Convolution performed by partially coherent system without averaging. **d,** Convolution performed by partially coherent system with 4-point average.

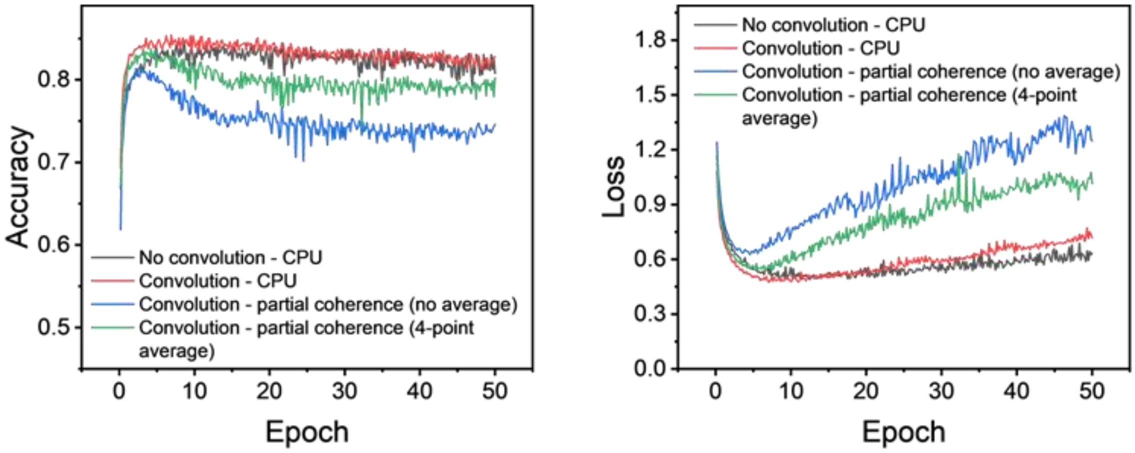

Figure.S19 Evolution of CNN loss and accuracy with increasing epochs in the classification of MNIST fashion products dataset.

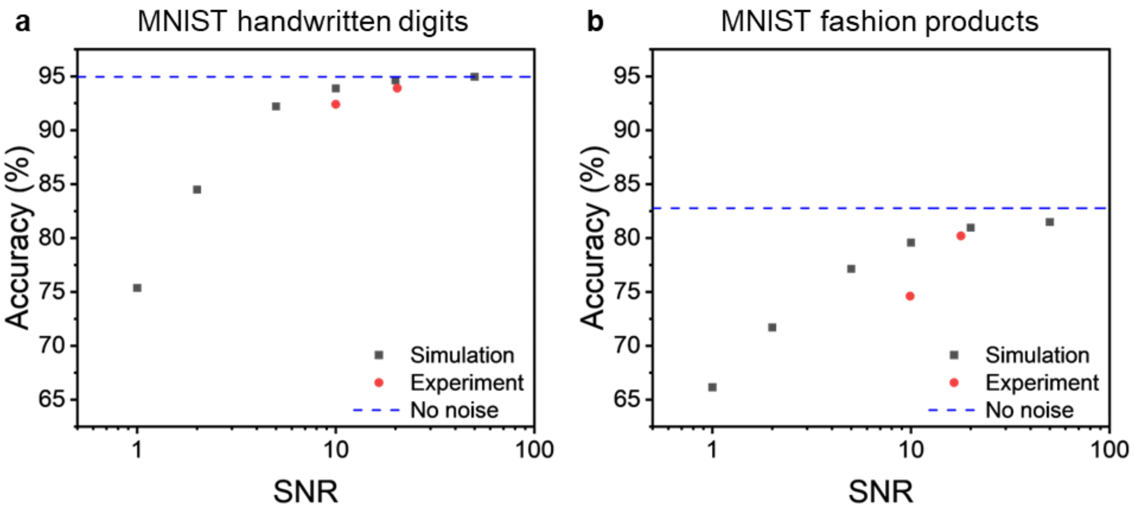

Figure.S20 Impact of SNR on classification accuracy. a, MNIST handwritten digits dataset. **b,** MNIST fashion products dataset.

-End of supplementary text 5

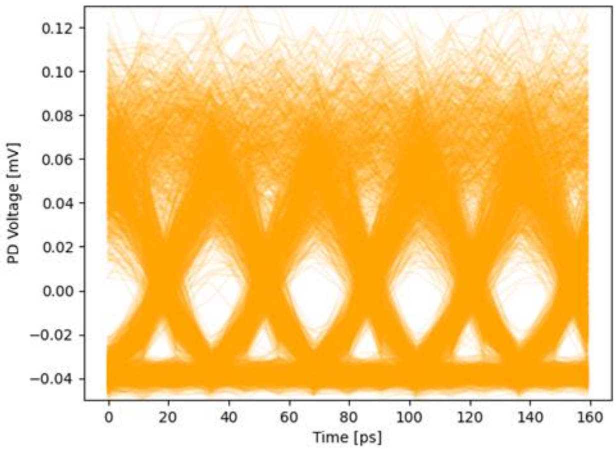

Figure.S21 Eye-diagram obtained from modulating partially coherent light at 30 GHz, limited by the electrical bandwidth of the receiver. In principle, the maximum data loading rate of the photonic EAM tensor core is 50 GHz, limited by the electrical bandwidth of IMEC EAM[3].

6. Limitation of partially coherent approach and comparison with coherent approach

Leveraging partially coherent light in a photonic tensor core enables the distribution of light within the same optical window across the entire core by eliminating phase fluctuation, thereby effectively enhancing data processing parallelism. On the contrary, utilizing coherent light necessitates distinct wavelengths for each input channel to circumvent interference. Given an N-input-channel photonic tensor core, an available total bandwidth of 100 nm (e.g., 1500 nm to 1600 nm), and a minimum 0.8-nm spacing between adjacent wavelength channels (aligned with the ITU grid for 50 GHz modulation), the coherent approach can offer a parallelism $P=(100/0.8/N)$, while the partially coherent approach provides a $P=100/OB$, independent of the number of input channels N, where OB is the optical bandwidth of partially coherent light. Meanwhile, the lower SNR inherent to filtered ASE partially coherent light should be considered. This SNR is dependent on both the optical bandwidth and the intensity received at the photodetector.

Under these assumptions and analyses, **Figure. S22** shows the dependency of parallelism and SNR on photonic tensor core size, optical bandwidth, and intensity received at the photodetector. At N=20, both the coherent and partially coherent approaches reach 4-bit resolution. The parallelism of coherent approach is 6, while the parallelism of partially coherent approach is 12 and 25 with OB of 8 nm and 4 nm respectively. The partially coherent approach exhibits more parallelism advantages for large photonic tensor cores ($N\geq42$). Within $42\leq N\leq62$, the coherent approach's parallelism is 2, contrasting with the partially coherent approach's parallelism of 6, 12, and 25 with OB of 16 nm, 8 nm, and 4 nm, respectively. For $63\leq N\leq125$, the parallelism of coherent approach diminishes to 1, and for $N>125$ it becomes inoperable. However, a partially coherent approach consistently preserves its parallelism. The limitations of partially coherent approach are related to the reduced SNR. For a high photodetector

receiving intensity of 3.33 mW, the SNR of coherent approach is higher than that of the partially coherent approach by 1-2 orders of magnitude, indicating superior computing accuracy. This SNR advantage diminishes to less than an order at a moderate 0.3 mW and becomes comparable at a lower 0.024 mW. Notably, in large-scale photonic tensor cores, the intensity received at the photodetector is usually compromised due to its distribution among numerous unit cells and accumulated insertion losses, resulting in a typical photodetector receiving intensity spanning 0.1 μW to 0.1 mW.

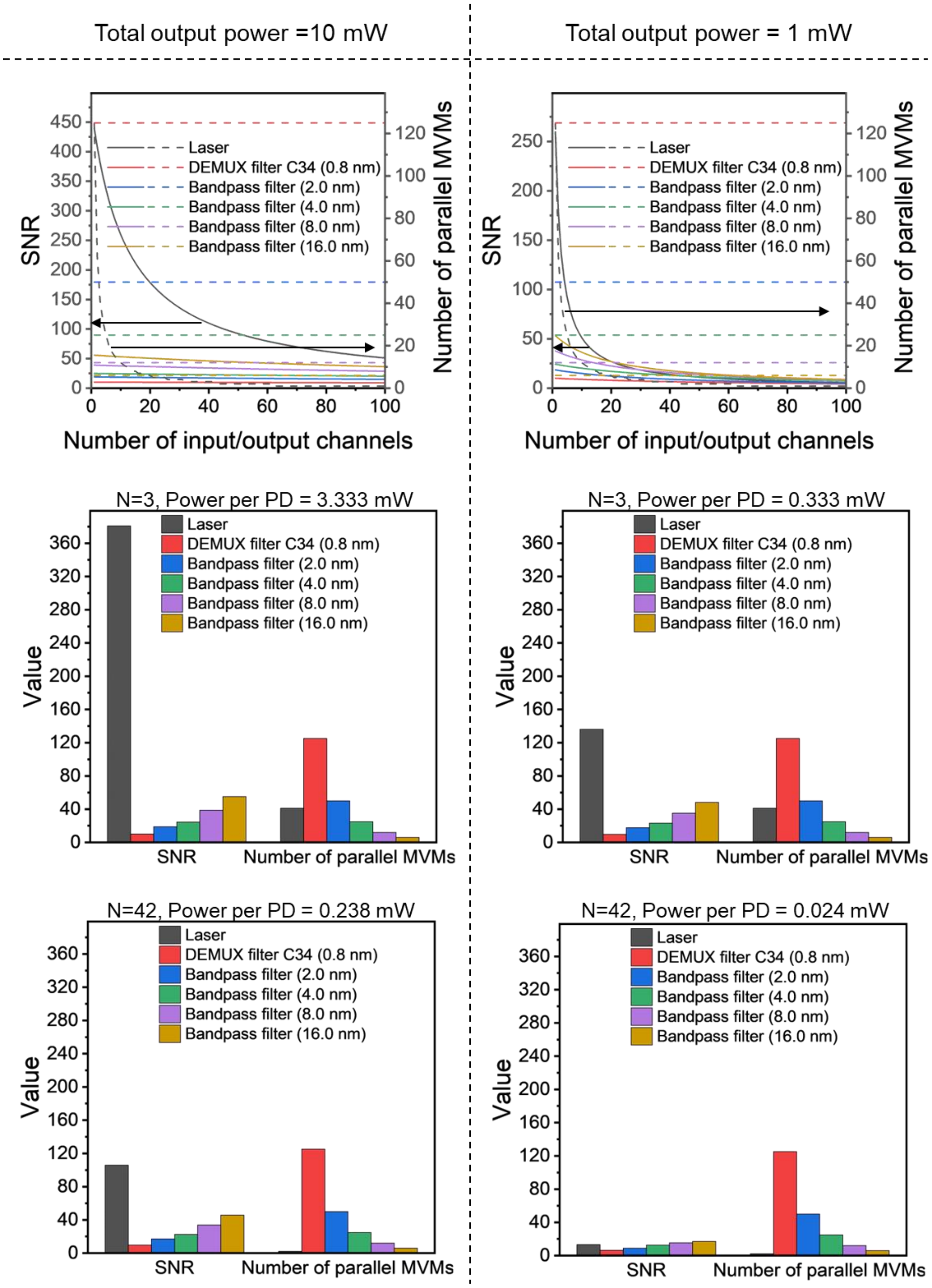

Figure.S22 Dependence of parallelism and SNR on the size of photonic tensor core, optical bandwidth, and intensity received at the photodetector.

In partially coherent systems, it is crucial to note that the requisite optical delay lines, employed to reduce phase sensitivity, can lead to very long waveguides. This could potentially result in a higher propagation loss and an additional footprint. To address this issue, we

introduce an architectural design depicted in **Figure. S23**, wherein all optical delay lines are coiled around the photonic tensor core, thereby achieving a high area efficiency.

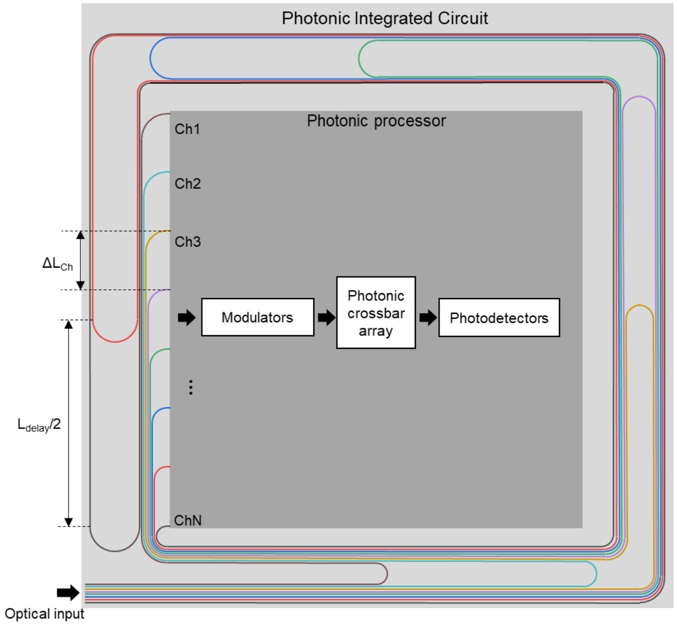

Figure.S23 Proposed architecture design for area-efficient optical delays.

Consider an $N \times N$ photonic tensor core, with a ΔL_{Ch} spacing between adjacent input channels (same ΔL_{Ch} for output channels). In the presented specific case of a single wraparound of the entire array, the perimeter amounts to $4\times(N\text{-}1)\times\Delta L_{Ch}$, resulting in the longest delay line being $8\times(N\text{-}1)\times\Delta L_{Ch}$ in length. The total area is computable, presuming a bend radius of 50 µm and a dense waveguide spacing of 5 µm. The path difference between adjacent delay lines is $2\times[4(N\text{-}1)\times\Delta L_{Ch}/N]\text{-}\Delta L_{Ch}$, which determines the minimum feasible optical bandwidth. A narrower optical bandwidth, implying a higher degree of coherence (refer to **Fig. 2e** in the main text), needs a longer delay line length. **Figure. S24** illustrates the dependencies of the longest delay line length, percentage area increase, and the minimal feasible optical bandwidth on the tensor core size and adjacent channel spacing. The longest delay line length typically exceeds 1 m for large photonic tensor core, and can reach 10 m for $N>100$ (**Figure. S24a**). With this regard, ultra-low-loss waveguides are necessary[8–10]. The percentage area increase remains below 1% (**Figure. S24b**), underscoring the high area efficiency of the proposed architecture. Wider optical bandwidths are achievable with reduced channel spacings (**Figure. S24c**).

Employing a channel spacing of 150 μm ensures an attainable optical bandwidth of 4 nm. Wider optical bandwidths can also be achieved by encircling the delay lines additional rounds around the array, incurring a negligible area penalty according to **Figure. S24b**.

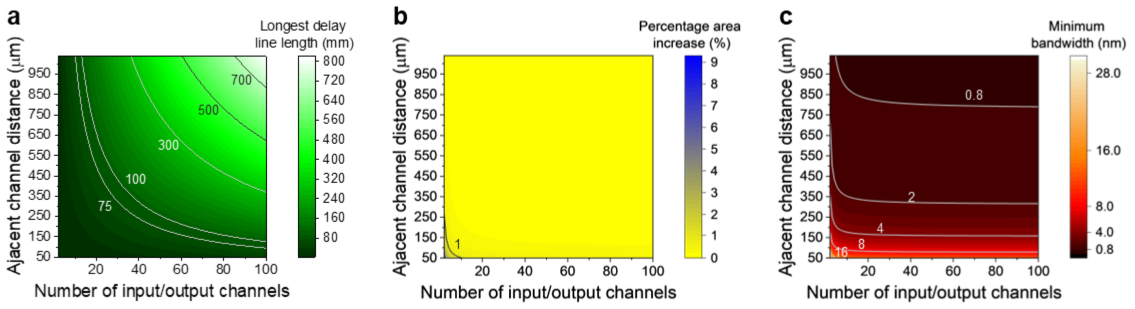

Figure.S24 Dependence of the longest delay line length, percentage area increase, and the minimum feasible optical bandwidth on the tensor core size and adjacent channel spacing.

7. Solutions to address the long delay line issue

While the footprint issue can be addressed by the design shown in **Fig. S23**, the long optical delay line issue still requires attention. Although ultralow loss Si_3N_4 waveguides of 0.1 dB/m[8] and 1 dB/m[9] have been reported, they are not foundry-available, which limits their scalability. Foundry-available silicon nitride-on-silicon platform is a feasible solution to address the long optical delay line issue[11]. The silicon nitride-on-silicon platform can harness the low-loss advantage of silicon nitride and active device availability of silicon. If we assume practically that the silicon nitride waveguide has a loss of 0.4 dB/cm[12]; and we further require the longest delay line to have a total loss less than 3 dB, the longest delay cannot exceed 75 mm. This 75-mm delay line limits the number of input channels to 59 if an optical bandwidth of 4 nm is needed (**Fig. S24a** and **Fig. S24c**). The presented numbers of channels and optical bandwidth are given as examples only. However, we note that this long delay line issue only exists if we assume the use of only one ASE source for the whole system. In practical implementations, an array of independent ASE sources working at the same wavelength can be employed, with each ASE source driving a few tens of input waveguide channels. These independent ASE sources are uncorrelated, eliminating the need for longer delay lines to overcome the coherence length of a single source. Waveguide integrated ASE sources have been demonstrated by a few groups[2,13]. From a system point of view, their integration into a photonic tensor core is technically similar to laser/waveguide integration. The schematic of a system with multiple ASE sources is presented in **Figure. S25.** Delay lines are sketched in spirals instead of wrapping around the processor for visual clarity. All ASE sources operate at the same optical band. The number of input channels driven by each ASE source is 59 for consistency with the example given above. This number can be adjusted depending on the waveguide loss, adjacent channel distance, and required optical bandwidth.

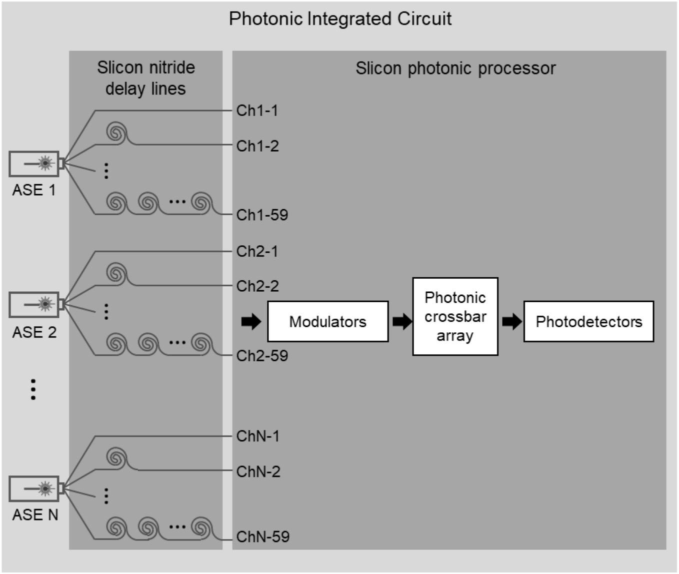

Figure.S25 Schematic of a system with multiple ASE sources. The total number of input channels in this schematic is 59×N, where N is the number of ASE sources.

-End of supplementary text 6

8. Comparison with state-of-the-art photonic computing systems

Table.S1 General comparison of architecture, modulation mechanism, sources of fluctuations, expected parallelism and SNR

	MZI mesh	MRR weight bank	Photonic tensor core (Comb)	**Photonic tensor core (Partial coherence)**
Size of matrix (inputs×outputs)	N×N	N×N	N×N	**N×N**
Modulated property	Phase	Phase	Amplitude	**Amplitude**
Number of modulated weights	N^2	N^2	N^2	$\mathbf{N^2}$
Sources of fluctuation on chip	Random phase fluctuation + Thermal crosstalk + Temperature fluctuation	Thermal crosstalk + Temperature fluctuation	Temperature fluctuation	**Temperature fluctuation**
Temperature sensitivity	0.013N π/K[14]	0.1 nm/K[15]	0.08 dB/K[16]	**0.08 dB/K**
Light source	Coherent frequency comb	Coherent frequency comb	Coherent frequency comb	**Partially coherent filtered ASE**
Parallelism @ N=125	100/BW[a]: 125 @ 0.8 nm BW	1[b]	1	**100/BW: 125 @ 0.8 nm BW 50 @ 2.0 nm BW 12 @ 8.0 nm BW**
SNR @ 2 GSa/s and 0.1 mW intensity at photodetector	51.0	51.0	51.0	**8.9 @ 0.8 nm BW 14.9 @ 2.0 nm BW 28.6 @ 8.0 nm BW**
SNR @ 2 Gsa/s and 0.01 mW intensity at photodetector	5.6	5.6	5.6	**4.3 @ 0.8 nm BW 5.0 @ 2.0 nm BW 8.3 @ 8.0 nm BW**

[a]Dispersion is not considered. The phase map designated for one wavelength could be distorted at a different wavelength. And the cumulative phase error can be large.

[b]We assume that 125 wavelength channels is possible for MRR weight bank, though it is expected that only 60 channels are available[17].

Our partially coherent approach uniquely features phase-insensitivity throughout the whole system, addressing the predominant challenge of phase fluctuation that hinders most large-scale photonic circuits, thus promising the potential to scaling up. The amplitude modulation for weight encoding in our approach has a low temperature sensitivity of 0.08 dB/K[16], which, if necessary, can be eliminated by a TEC controller. The adoption of partial coherence enables the distribution of light within the same optical window across the entire photonic processor while preserving multiplexing capability. Consequently, it achieves high parallelism,

comparable to MZI meshes, and superior to MRR weight banks and coherent comb-based photonic tensor cores. While the theoretical scalability of the MZI mesh is acknowledged, practical upscaling is impeded by accumulative random phase fluctuation and thermal crosstalk, which present formidable challenges for mitigation. A notable limitation of the partially coherent approach lies in its lower SNR, intrinsically linked to the stochastic property of the ASE light source. At an elevated optical power (> 1 mW received at the photodetector), the coherent approach provides markedly higher SNR than their partially coherent counterparts. However, at an intensity received by the photodetector ranging from 0.1 µW to 0.1 mW, which is the range of interest to many applications, the SNRs become comparable. Improved SNR could potentially be realized by replacing EDFA ASE with broadband superluminescent diodes (SLED)[18] and further coupling SLEDs with saturated semiconductor optical amplifiers (SOA) to suppress noise[19].

Table.S2 Performance comparison with the-state-of-the-art works

Ref	Year	Technology	Data loading rate (GSa/s)	System processing speed (TOPS)	Throughput per optical carrier (TOPS)	Energy efficiency (TOPS/W),
[20]	2023	MRR weight bank + Comb	17	1.36×10^{-1}	3.4×10^{-2}	0.2
[21]	2023	Multimode interference	16.6	2.65×10^{-1}	6.63×10^{-2}	0.2
[22]	2022	MRR weight bank	20	4.8×10^{-1}	1.2×10^{-1}	N.A.
[23]	2022	MZI mesh + diffractive cell	1×10^{-5}	2×10^{-6}	2×10^{-6}	1.1×10^{-4} (light source, electronics, and receiver not considered)
[24]	2022	PIN attenuator array	22 (estimated)	2.07 (estimated)	2.07 (estimated)	2.9 (estimated)
[25]	2021	Phase change material+Comb	2	2.56×10^{-1}	6.4×10^{-2}	0.4
[26]	2021	MRR weight bank	0.375	6×10^{-3}	1.5×10^{-3}	N.A.
[27]	2021	MZI mesh	1×10^{-5}	6.4×10^{-7}	6.4×10^{-7}	5.72×10^{-7} (light source, electronics, and receiver not considered)
[28]	2020	SOA array	10	8×10^{-2}	2×10^{-2}	0.24 (electronics and receiver not considered)
**This work**	**2023**	**Partially coherent system**	**2**	**1.08×10^{-1}**	**1.08×10^{-1}**	**1 (estimated)**

-End of supplementary text 7

Table.S3 Clinical information of ten patients

**Subject Number**	**Gender**	**Age**	**Height (m)**	**Weight (kg)**	**Unified Parkinson's Disease Rating Scale***
1	Female	77	1.58	67	N.A.
2	Male	73	1.70	70	27
3	Female	54	1.66	65	N.A.
4	Male	63	1.74	80	21
5	Female	64	1.67	55	44
6	Male	52	1.65	75	42
7	Male	60	1.65	65	46
8	Male	50	1.73	69	41
9	Male	64	1.65	80	36
10	Male	57	1.60	88	48

*Unified Parkinson's Disease Rating Scale is a rating tool used to gauge the severity and progression of Parkinson's disease. It is a gold standard used by neurologists for monitoring the response to medications used to decrease the signs and symptoms of Parkinson's disease.

References

1. Becker, M. *et al.* Activation Functions in Non-Negative Neural Networks. in *Neural Information Processing Systems* (2023). doi:10.33564/ijeast.2020.v04i12.054.

2. Mehta, K. *et al.* High-Power Heterogeneously Integrated III-V/Silicon Superluminescent Diode. *IEEE Photonics Technol. Lett.* **35**, 365–368 (2023).

3. Heyn, P. De *et al.* High-Speed Germanium-Based Waveguide Electro-Absorption Modulator. in *2016 21st OptoElectronics and Communications Conference (OECC) held jointly with 2016 International Conference on Photonics in Switching (PS)* WD1-1 (2016).

4. Moazeni, S. *et al.* A 40-Gb/s PAM-4 transmitter based on a ring-resonator optical DAC in 45-nm SOI CMOS. *IEEE J. Solid-State Circuits* **52**, 3503–3516 (2017).

5. Saeedi, S. & Emami, A. A 25Gb/s 170μW/Gb/s optical receiver in 28nm CMOS for chip-to-chip optical communication. in *2014 IEEE Radio Frequency Integrated Circuits Symposium* (2014). doi:10.1109/RFIC.2014.6851720.

6. Kull, L. *et al.* A 10-Bit 20-40GS/s ADC with 37dB SNDR at 40GHz input using first order sampling bandwidth calibration. in *IEEE Symposium on VLSI Circuits, Digest of Technical Papers* 275–276 (IEEE, 2018). doi:10.1109/VLSIC.2018.8502268.

7. Jouppi, N. P. *et al.* Ten lessons from three generations shaped Google's TPUv4i: Industrial product. in *2021 ACM/IEEE 48th Annual International Symposium on Computer Architecture (ISCA)* (IEEE, 2021). doi:10.1109/ISCA52012.2021.00010.

8. Bauters, J. F. *et al.* Planar waveguides with less than 0.1 dB/m propagation loss fabricated with wafer bonding. *Opt. Express* **19**, 24090–24101 (2011).

9. Liu, J. *et al.* High-yield, wafer-scale fabrication of ultralow-loss, dispersion-engineered silicon nitride photonic circuits. *Nat. Commun.* **12**, 2236 (2021).

10. Torres-Company, V., Ye, Z., Zhao, P., Karlsson, M. & Andrekson, P. A. Ultralow-loss silicon nitride waveguides for parametric amplification. in *Optical Fiber Communication (OFC) Conference* W4J.3 (OSA, 2022).

11. Sacher, W. D. *et al.* Monolithically Integrated Multilayer Silicon Nitride-on-Silicon Waveguide Platforms for 3-D Photonic Circuits and Devices. *Proc. IEEE* **106**, 2232–2245 (2018).

12. Siew, S. Y. *et al.* Review of Silicon Photonics Technology and Platform Development. *J. Light. Technol.* **39**, 4374–4389 (2021).

13. De Groote, A. *et al.* Heterogeneously integrated III–V-on-silicon multibandgap superluminescent light-emitting diode with 290 nm optical bandwidth. *Opt. Lett.* **39**, 4784 (2014).

14. Guan, X., Wang, X. & Frandsen, L. H. Optical temperature sensor with enhanced sensitivity by employing hybrid waveguides in a silicon Mach-Zehnder interferometer. *Opt. Express* **24**, 16349–16356 (2016).

15. Lima, T. F. De *et al.* Design automation of photonic resonator weights. *Nanophotonics* **11**, 3805–3822 (2022).

16. Fujikata, J. *et al.* High-performance Ge/Si electro-absorption optical modulator up to 85°C and its highly efficient photodetector operation. *Opt. Express* **31**, 10732–10743 (2023).

17. Preston, K., Sherwood-Droz, N., Levy, J. S. & Lipson, M. Performance guidelines for WDM interconnects based on silicon microring resonators. in *Conference on Lasers*

and Electro-Optics (CLEO) CThP4 (2011).

18. Guo, X. *et al.* Correlation between emission and relative intensity noise spectral profiles of an Er-doped fiber superfluorescent source. *AIP Adv.* **12**, 055226 (2022).

19. Zhao, M., Morthier, G. & Baets, R. Analysis and Optimization of Intensity Noise Reduction in Spectrum-Sliced WDM Systems Using a Saturated Semiconductor Optical Amplifier. *IEEE Photonics Technol. Lett.* **14**, 390–392 (2002).

20. Bai, B. *et al.* Microcomb-based integrated photonic processing unit. *Nat. Commun.* **14**, 66 (2023).

21. Meng, X. *et al.* Compact optical convolution processing unit based on multimode interference. *Nat. Commun.* **14**, 3000 (2023).

22. Xu, S., Wang, J., Yi, S. & Zou, W. High-order tensor flow processing using integrated photonic circuits. *Nat. Commun.* **13**, 7970 (2022).

23. Zhu, H. H. *et al.* Space-efficient optical computing with an integrated chip diffractive neural network. *Nat. Commun.* **13**, 1044 (2022).

24. Ashtiani, F., Geers, A. J. & Aflatouni, F. An on-chip photonic deep neural network for image classification. *Nature* **606**, 501–506 (2022).

25. Feldmann, J. *et al.* Parallel convolutional processing using an integrated photonic tensor core Supplementary Materials for Parallel convolution processing using an integrated photonic tensor core. **589**, (2021).

26. Huang, C. *et al.* A silicon photonic–electronic neural network for fibre nonlinearity compensation. *Nat. Electron.* **4**, 837–844 (2021).

27. Zhang, H. *et al.* An optical neural chip for implementing complex-valued neural

network. *Nat. Commun.* **12**, 457 (2021).

28. Shi, B., Calabretta, N. & Stabile, R. Deep Neural Network through an InP SOA-Based Photonic Integrated Cross-Connect. *IEEE J. Sel. Top. Quantum Electron.* **26**, 7701111 (2020).
